# FOXP4 promotes metastatic progression in colorectal cancer through transcriptional activation of BAG3

**DOI:** 10.1038/s41419-026-08941-5

**Published:** 2026-06-11

**Authors:** Jiayun Wang, Teng Yu, Lin Liu, Kaiyuan Hui, Chunluan Yuan, Zien Yu, Yilin Wang, Xiuming Li, Xiaozhu Shen, Xiaodong Jiang, Bin Liu

**Affiliations:** 1 Jiangsu Key Laboratory of Marine Pharmaceutical Compound Screening, College of Pharmacy, Jiangsu Ocean University, Lianyungang, China; 2 Department of Pathology, Shanghai Ruijin Hospital, Shanghai Jiao Tong University School of Medicine, Shanghai, China; 3 Department of Oncology, Lianyungang Clinical College of Nanjing Medical University/The First People’s Hospital of Lianyungang, Lianyungang, China; 4 Department of Oncology, The Affiliated Lianyungang Hospital of Xuzhou Medical University/The First People’s Hospital of Lianyungang, Lianyungang, China; 5 Department of Geriatrics, Lianyungang Second People’s Hospital, Lianyungang, China

**Keywords:** Mechanisms of disease, Cancer screening

## Abstract

Metastatic dissemination remains a major cause of mortality in colorectal cancer (CRC), yet the transcriptional basis of epithelial plasticity is not fully understood. Here, we identified Forkhead Box P4 (FOXP4) as a transcription factor associated with CRC progression. Integrated analyses across multiple patient cohorts, together with single-cell transcriptomic data, showed that FOXP4 is preferentially upregulated in malignant epithelial cells and is associated with poor patient survival. FOXP4 depletion impaired malignant phenotypes in vitro and reduced tumor growth, angiogenesis, and liver metastatic burden in vivo. Mechanistically, FOXP4 bound to the promoter region of the co-chaperone BAG3 and regulated its transcription. Consistent with this, BAG3 restoration or silencing partially reversed the phenotypic changes induced by FOXP4 perturbation. These findings suggest that FOXP4 promotes EMT-associated cellular plasticity, at least in part, through BAG3. Together, these results support a FOXP4–BAG3 regulatory axis linked to EMT-related transcriptional programs in CRC. This study provides additional insight into the molecular basis of epithelial plasticity in CRC and supports further investigation of this pathway.

## Introduction

The metastatic dissemination of cancer cells is the primary cause of mortality in patients with colorectal cancer (CRC), a disease that continues to impose a formidable global health burden [1, 2]. This metastatic cascade is a complex, multistep process that depends on substantial cellular plasticity [3]. A major manifestation of this plasticity is the epithelial-mesenchymal transition (EMT), a latent developmental program co-opted by cancer cells to acquire migratory and invasive capabilities, stem-like properties, and profound therapeutic resistance [4–7]. EMT is regulated by a set of key transcription factors (TFs), including members of the SNAIL, ZEB, and TWIST families, which repress epithelial genes such as CDH1 (E-cadherin) while activating a mesenchymal gene expression program [8–11]. While these EMT-TFs are the established downstream effectors, the upstream signaling pathways and the broader transcriptional networks that dictate their expression and activity in the tumor microenvironment are complex and remain an area of intense investigation [12–15]. Identifying additional upstream regulators that contribute to EMT-associated programs may therefore help refine the mechanistic understanding of CRC progression and suggest new therapeutic directions.

Among the families of TFs, the Forkhead Box (FOX) proteins play important roles in cell fate during embryogenesis, tissue homeostasis, and tumorigenesis [16–18]. This functionally diverse family, characterized by a “winged-helix” DNA-binding domain, includes prominent oncogenes like FOXM1, an important regulator of the cell cycle [19–23], as well as potent tumor suppressors like the FOXO subfamily, which integrate metabolic and stress signals to control apoptosis and cell cycle arrest [24–26]. In the context of CRC, the dysregulation of various FOX proteins has been implicated in disease progression. For instance, the FOXP subfamily itself includes FOXP3, the lineage-defining TF for regulatory T cells, whose function within the tumor microenvironment profoundly impacts anti-tumor immunity [27–29]. This context-dependent and often opposing functionality of FOX family members underscores the necessity of characterizing each member’s specific role within a given cancer type [30].

Concurrently, the co-chaperone BCL2-associated athanogene 3 (BAG3) has emerged as a key pro-survival factor enabling cancer cells to withstand proteotoxic stress [31–33]. While BAG3 has been reported to regulate the stability of proteins involved in tumor progression [34, 35], the upstream transcriptional mechanisms responsible for its dysregulation in CRC remain unclear.

Our previous work identified FOXP4 as an oncogenic factor in ovarian cancer [36], positioning it as a transcription factor of interest. Given the established importance of both the FOX protein family and the pro-tumorigenic functions of BAG3, we sought to investigate a potential connection in CRC, where our initial screening suggested that both factors were upregulated in CRC. We hypothesized that FOXP4 may contribute to CRC progression through transcriptional regulation of BAG3 and related EMT-associated programs. In the present study, we investigated the role of FOXP4 in colorectal cancer progression and explored its connection with epithelial–mesenchymal transition (EMT)-associated programs. We identified BAG3 as a transcriptional target of FOXP4 and examined the functional significance of the FOXP4–BAG3 axis in colorectal cancer cells. Our findings support a model in which FOXP4 contributes to EMT-associated phenotypes through transcriptional regulation of BAG3, thereby providing mechanistic insight into epithelial plasticity in colorectal cancer.

## Materials and methods

### Bioinformatic analysis

Publicly available microarray datasets GSE23878, GSE142279, GSE33113, GSE227550, GSE166555, GSE242332 and GSE103475 were downloaded from the Gene Expression Omnibus (GEO) database (https://www.ncbi.nlm.nih.gov/geo/). Differential expression analysis between tumor and normal tissues or between experimental groups was performed using the limma package in R. Genes with a *p*-value < 0.05 and |log2(Fold Change)| >1.2 were considered differentially expressed genes (DEGs). Heatmaps and volcano plots were generated using the pheatmap and ggplot2 packages, respectively. The human transcription factor list was obtained from the Human Transcription Factors database (http://humantfs.ccbr.utoronto.ca/). Venn diagrams were generated using the VennDiagram package in R. RNA-sequencing data and clinical information for the Colon Adenocarcinoma (COAD) cohort were downloaded from The Cancer Genome Atlas (TCGA) portal via the GDC Data Portal. Survival analysis was performed using the GEPIA2 web server (http://gepia2.cancer-pku.cn/) and the UALCAN portal (https://ualcan.path.uab.edu/). Gene Set Enrichment Analysis (GSEA) was performed using the GSEA software (v4.1.0, Broad Institute) with the Hallmark gene sets (h.all.v7.4.symbols.gmt). Co-expression analysis was performed using COXPRESdb (https://coxpresdb.jp/) and analysis of TCGA-COAD data. Potential FOXP4 target genes were predicted using the ChIP-Atlas database (https://chip-atlas.org/). Single-cell RNA-sequencing data from GSE166555 were analyzed using the Seurat package in R, and EMT module scores were calculated using previously reported EMT gene signatures. Copy number variation (CNV) data were obtained from TCGA and analyzed using GISTIC2.0-defined categories. EMT module scores were calculated based on previously reported EMT gene signatures.

### Cell culture and transfection

The human colorectal adenocarcinoma cell lines HCT116 and SW480 were obtained from the American Type Culture Collection (ATCC, Manassas, VA, USA) and authenticated via short tandem repeat (STR) profiling prior to use. HCT116 cells were cultured in McCoy’s 5 A medium (KGL1701-500, KeyGEN BioTECH), and SW480 cells were maintained in Iscove’s Modified Dulbecco’s Medium (IMDM) (KGL1101-500, KeyGEN BioTECH), both supplemented with 10% Fetal Bovine Serum (FBS, Gibco, Cat# A5669701) and 1% Penicillin–Streptomycin (Gibco, Cat# 15140122). All cells were maintained in a humidified incubator at 37 °C with 5% CO2.

For transient transfection, plasmids were transfected into cells using Lipofectamine 3000 Reagent (Invitrogen, Cat# L3000015) according to the manufacturer’s instructions. For stable knockdown, lentiviral particles encoding shRNAs targeting FOXP4 (shFOXP4 #1, #2) or a non-targeting control (pLKO.1) were purchased from Sigma-Aldrich (MISSION shRNA). Lentiviral transduction was performed in the presence of 8 μg/mL polybrene (Sigma-Aldrich, Cat# H9268). Stably transduced cells were selected and maintained in medium containing 2 μg/mL puromycin (Gibco, Cat# A1113803).

### Plasmids and reagents

The human FOXP4 overexpression plasmid (pcDNA3.1-FOXP4) and BAG3 overexpression plasmid were synthesized by GeneCopoeia (Guangzhou, China). The pLKO.1-puro empty vector was used as a control. shRNAs targeting human FOXP4 (TRCN0000018533, TRCN0000018534) and BAG3 (TRCN0000148729) were obtained from the MISSION shRNA library (Sigma-Aldrich).

### RNA extraction and quantitative real-time PCR (qRT-PCR)

Total RNA was extracted from cells using TRIzol (Invitrogen, Cat# 15596026CN). cDNA was synthesized from 1 μg of total RNA using the PrimeScript™ RT Reagent Kit with gDNA Eraser (Takara, Cat# RR047A). qRT-PCR was performed on a QuantStudio 5 Real-Time PCR System (Applied Biosystems) using TB Green® Premix Ex Taq™ II (Tli RNaseH Plus) (Takara, Cat# RR820A). The relative expression of target genes was calculated using the 2^–ΔΔCt^ method, with GAPDH serving as the internal control.

### Western blot analysis

Cells were lysed in RIPA buffer (Beyotime, Cat# P0013B) supplemented with a protease and phosphatase inhibitor cocktail (Roche, Cat# 04693132001, 04906837001). Protein concentration was determined using the BCA Protein Assay Kit (Thermo Fisher Scientific, Cat# 23225). Equal amounts of protein (30 μg) were separated by 10% SDS-PAGE and transferred to polyvinylidene difluoride (PVDF) membranes (Millipore, Cat# IPVH00010). The membranes were blocked with 5% non-fat milk in Tris-buffered saline with 0.1% Tween 20 (TBST) for 1 h at room temperature, then incubated overnight at 4 °C with primary antibodies. The primary antibodies used were: anti-FOXP4 (1:1000, Abcam, Cat# ab242127), anti-BAG3 (1:1000, Cell Signaling Technology, Cat# 23842), anti-E-cadherin (1:1000, Cell Signaling Technology, Cat# 3195), anti-N-cadherin (1:1000, Cell Signaling Technology, Cat# 13116), anti-Vimentin (1:1000, Cell Signaling Technology, Cat# 5741), anti-ZEB1(1:1000, Cell Signaling Technology, Cat# 70512), anti-β-catenin (1:1000, Cell Signaling Technology, Cat# 8480), and anti-GAPDH (1:5000, Proteintech, Cat# 60004-1-Ig). After washing, membranes were incubated with HRP-conjugated secondary antibodies (1:1000, Cell Signaling Technology, Cat# 7074 or 7076) for 1 h. Protein bands were visualized using an enhanced chemiluminescence (ECL) detection kit (Thermo Scientific, Cat# 32106) and imaged with a ChemiDoc XRS+ System (Bio-Rad).

### Cell proliferation, colony formation, and EdU assays

For the cell proliferation assay, 5 × 10^3^ cells per well were seeded in 96-well plates. Cell viability was measured at 24, 48, and 72 h using the Cell Counting Kit-8 (CCK-8, Dojindo, Cat# CK04). For the colony formation assay, 500 cells per well were seeded in 6-well plates and cultured for 10–14 days. Colonies were fixed with 4% paraformaldehyde, stained with 0.1% crystal violet (Sigma-Aldrich, Cat# V5265), and counted. For the EdU assay, cell proliferation was assessed using the Click-iT™ EdU Imaging Kit (Invitroge, Catalog #C10337) according to the manufacturer’s protocol. Images were captured with a fluorescence microscope (Nikon).

### Migration, invasion and wound-healing assays

Cell migration and invasion were assessed using 24-well Transwell chambers with 8.0 μm pore size inserts (Corning, Cat#3422). For the invasion assay, the inserts were pre-coated with Matrigel (Corning, Cat# CLS356234). 5 × 10^4^ cells in serum-free medium were added to the upper chamber, and the lower chamber was filled with DMEM containing 20% FBS. After 24 h (migration) or 48 h (invasion), cells on the upper surface of the membrane were removed. Cells that had migrated or invaded the lower surface were fixed, stained with 0.1% crystal violet, and counted in five random fields under a microscope. For the wound-healing assay, cells were seeded in 6-well plates and grown to confluence. A scratch was made with a 100 μL pipette tip. Images were captured at 0,24 and 48 h, and the wound closure area was measured using Image J software.

### Chromatin immunoprecipitation (ChIP) assay

ChIP assays were performed using the SimpleChIP^®^ Plus Enzymatic Chromatin IP Kit (Magnetic Beads) (Cell Signaling Technology, Cat# 9005) following the manufacturer’s instructions. Briefly, cells were cross-linked with 1% formaldehyde and lysed. Chromatin was digested with micrococcal nuclease and sonicated. The chromatin solution was then immunoprecipitated overnight at 4 °C with 5 μg of anti-FOXP4 antibody (Abcam, Cat# ab242127) or normal rabbit IgG (Cell Signaling Technology, Cat# 2729) as a negative control. The immunoprecipitated DNA was purified and analyzed by qRT-PCR.

### Chick chorioallantoic membrane (CAM) assay

Fertilized specific-pathogen-free (SPF) chicken eggs were purchased from a local hatchery and incubated at 37 °C with 60% humidity. On embryonic day 3 (EDD3), a window was opened in the shell. On EDD8, 2 × 10^6^ cells resuspended in 30 μL of Matrigel were grafted onto the CAM. On EDD14, the tumors and surrounding CAM were imaged in situ. The tumors were then excised, photographed, and weighed. All procedures were performed under aseptic conditions to minimize contamination and ensure reproducibility.

### Murine orthotopic colorectal cancer model

BALB/c nude mice were housed under a 12-h light/dark cycle with free access to food and water. After acclimatization, mice were randomly assigned to the indicated experimental groups. HCT116 or SW480 cells (1 × 10^6^), stably transduced with luciferase and the indicated shRNA or expression vectors, were suspended in PBS/Matrigel (1:1) and surgically injected into the cecal wall. Tumor progression was monitored weekly by bioluminescence imaging using an IVIS Spectrum system (PerkinElmer). Blinding was applied during outcome assessment. Investigators responsible for image quantification and histological evaluation were blinded to group allocation until completion of the analysis.

### Immunohistochemistry

The colorectal adenocarcinoma specimens were collected during surgery at Ruijin Hospital, Shanghai Jiao Tong University School of Medicine. Serial 4-μm sections were obtained from 10% formalin-fixed paraffin-embedded (FFPE) specimens using a rotary paraffin microtome. Immunohistochemical (IHC) staining utilised a primary antibody against FOXP4 (16772-1-AP, Proteintech, 1:400). The IHC analysis was performed using the LEICA BOND-III automated staining platform. Whole slide images were acquired using a digital pathology scanner (NanoZoomer S210). Protein expression levels were evaluated using a semi-quantitative H-Score system (range: 0–300), calculated as follows: H-Score = ∑ (staining intensity × percentage of positive cells) (intensity grading: 0 = negative, 1 = weak positive, 2 = moderate positive, 3 = strong positive). For quality control, two pathologists independently scored the slides in a double-blind manner, and discrepant cases were reviewed by a third party and confirmed through a consensus meeting to determine the final results.

### Liver metastasis model

A liver metastasis model was established by intrasplenic injection of HCT116 or SW480 cells. After acclimatization, mice were randomly assigned to the indicated groups. Briefly, 2 × 10^6^ cells in 50 μL PBS were injected into the splenic parenchyma through a small left upper abdominal incision. Splenectomy was performed approximately 10 min later to prevent splenic primary tumor growth. Thirty days after implantation, mice were sacrificed, and the livers were excised for ex vivo bioluminescence imaging, gross photography, and counting of visible metastatic nodules. Liver image analysis, metastatic nodule counting, and histological assessment were performed in a blinded manner.

### Statistical analysis

All quantitative experiments were performed with at least three biological replicates. No animals or samples were excluded from the analysis. Sample size was not predetermined by formal statistical power analysis but was chosen based on previous experience and published studies using similar models. Data are presented as mean ± SD. Statistical analyses were performed using GraphPad Prism 10.1 (GraphPad Software, La Jolla, CA, USA). Two-tailed Student’s *t* test was used for comparisons between two groups, and one-way ANOVA followed by Tukey’s post hoc test was used for multiple-group comparisons. Survival was analyzed by the Kaplan–Meier method with the log-rank test. A *P* value < 0.05 was considered statistically significant (*P* < 0.05, *P* < 0.01, *P* < 0.001, *P* < 0.0001).

### Ethical approval

All animal experiments were conducted in accordance with the guidelines and regulations set forth by the Experiment Ethics Review Committee of Jiangsu Ocean University and were approved by this committee (Approval No. Jou2025051508). The use of human tissue samples in this study was approved by the Ethics Committee of Ruijin Hospital, Shanghai Jiao Tong University School of Medicine (Approval No. KY2025022).

## Results

### FOXP4 is a consistently upregulated transcription factor in colorectal cancer

**Fig. 1:**
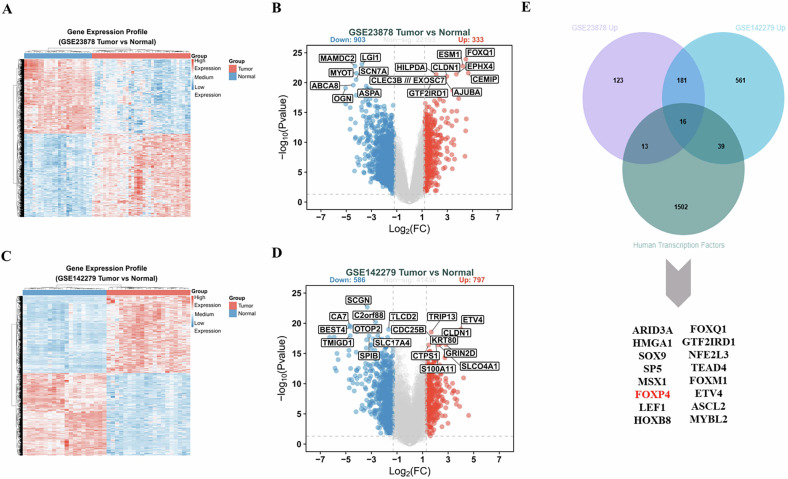
Identification of FOXP4 as a key upregulated transcription factor in colorectal cancer. Heatmap (**A**) and volcano plot (**B**) of differentially expressed genes (DEGs) identified from the GSE23878 dataset, comparing colon tumor (Tumor) tissues to adjacent normal (Normal) tissues. The analysis revealed 333 upregulated and 903 downregulated genes. Heatmap (**C**) and volcano plot (**D**) of DEGs from the independent GSE142279 dataset, confirming the expression patterns observed in GSE23878. This analysis identified 797 upregulated and 586 downregulated genes. In all heatmaps, red indicates high expression and blue indicates low expression. In volcano plots, red dots represent upregulated genes, blue dots represent downregulated genes, and gray dots represent non-significantly expressed genes. The cutoff for significant differential expression was set at a p-value < 0.05 and |log2 Fold Change | > 1.2. **E** Venn diagram illustrating the intersectional analysis to identify candidate oncogenic transcription factors. The analysis shows the overlap between upregulated genes from GSE23878, upregulated genes from GSE142279, and a comprehensive set of human transcription factors. This stringent filtering yielded 16 common transcription factors that are consistently overexpressed in colorectal cancer. Forkhead Box P4 (FOXP4), highlighted in red, was selected from this list for further investigation.

To identify key transcriptional regulators potentially driving colorectal cancer (CRC) progression, we initiated our investigation by performing a comprehensive transcriptomic analysis of independent, publicly available patient cohorts. We first analyzed two separate microarray datasets, GSE23878 and GSE142279, to robustly identify differentially expressed genes (DEGs) between CRC tumor tissues and adjacent normal colon tissues. Using a stringent cutoff (*p* < 0.05, |log2FC | > 1.2), we identified 333 upregulated and 903 downregulated genes in the GSE23878 dataset (*n* = 35 tumor, *n* = 24 normal; Fig. 1A, B). This differential expression pattern was validated in the independent GSE142279 cohort (*n* = 19 tumor, *n* = 19 normal), where we found 797 upregulated and 586 downregulated genes, confirming a consistent transcriptomic dysregulation signature in CRC (Fig. 1C, D).

Given that transcription factors (TFs) are master regulators of cellular programs and their aberrant activity is a hallmark of oncogenesis, we hypothesized that TFs consistently overexpressed in CRC may function as critical drivers of the disease. To pinpoint such high-confidence candidates, we employed a stringent filtering strategy. We performed an intersectional analysis between the upregulated gene sets from both GSE23878 (333 genes) and GSE142279 (797 genes) and a curated database of 1570 established human transcription factors. This multi-layered approach identified a core set of 16 TFs that were significantly and consistently upregulated across both independent CRC patient cohorts (Fig. 1E). Notably, this candidate list included several transcription factors with previously reported roles in CRC progression, such as LEF1, ASCL2, SOX9, MYBL2, and FOXM1, supporting the biological relevance of our screening approach. Among these candidates, Forkhead Box P4 (FOXP4) was selected for further investigation. Our previous work identified FOXP4 as an oncogenic factor in ovarian cancer and showed that it promotes tumor progression through PTK7-mediated activation of the Wnt/β-catenin pathway. Given our established expertise and the fundamental importance of this transcription factor in another malignancy, its consistent and significant upregulation in colorectal cancer immediately positioned it as a high-priority candidate for our current investigation.

Therefore, building upon our prior work, we selected FOXP4 from the candidate list to determine if it plays a similar oncogenic role in the context of colorectal cancer. We therefore selected FOXP4 for further investigation in CRC and examined whether it exerts a similar pro-tumorigenic role in this context.

### FOXP4 is overexpressed in colorectal cancer and is associated with poor prognosis

**Fig. 2:**
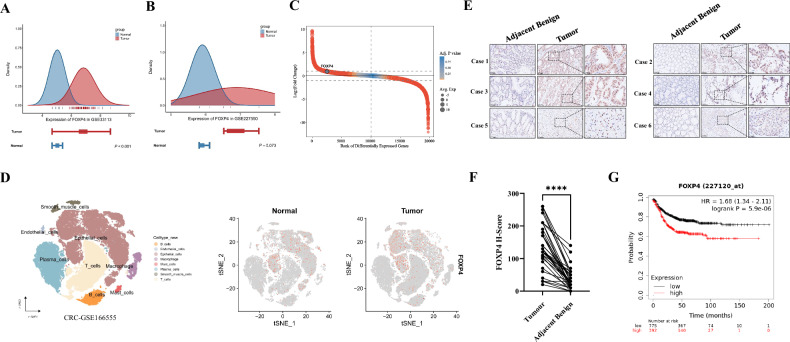
FOXP4 is overexpressed in colorectal cancer and is associated with poor prognosis. Kernel density plots showing the relative mRNA expression of FOXP4 in colon tumor versus normal tissues from two independent GEO datasets, GSE33113 (*n* = 90 tumor, *n* = 6 normal) (**A**) and GSE227550 (*n* = 3 tumor, *n* = 3 normal) (**B**). **C** Rank-based plot analysis of FOXP4 mRNA expression (Transcripts Per Million, TPM) in primary colon adenocarcinoma (COAD) tumors (*n* = 286) and adjacent normal tissues (*n* = 41) from The Cancer Genome Atlas (TCGA) database. **D** Single-cell RNA-sequencing analysis of a CRC patient cohort (GSE166555). Left: UMAP plot showing major cell lineages identified in the tumor microenvironment. Right: UMAP feature plot showing the specific expression of FOXP4, with the color scale indicating the level of expression. Expression is predominantly localized to the malignant cell cluster. **E** Immunohistochemical (IHC) staining for FOXP4 in six representative colorectal adenocarcinoma cases. **F** Quantification of FOXP4 protein levels. Box plots displaying H-scores of FOXP4 immunohistochemical staining in paired adjacent normal and tumor tissues. Statistical significance was assessed via a paired Student’s *t* test. **G** Kaplan-Meier survival curve for overall survival (OS) of a combined cohort of CRC patients (*n* = 1167), stratified by high (red line) and low (black line) FOXP4 expression (probe: 227120_at). The hazard ratio (HR), 95% confidence interval, and log-rank p-value are indicated. Data in (**A, B, E**) are presented as mean ± SD. Statistical significance was determined by a two-tailed Student’s *t* test. *p < 0.05, **p < 0.01, ****p < 0.0001.

Having identified FOXP4 as a consistently upregulated transcription factor in CRC through our initial screening, we next sought to validate its expression pattern and assess its clinical significance using multiple independent cohorts and methodologies. First, we re-analyzed two additional microarray datasets from the Gene Expression Omnibus (GEO) database. In the GSE33113 dataset, which includes a substantial number of samples, the mRNA expression of FOXP4 was significantly elevated in tumor tissues (*n* = 90) compared to normal colon tissues (*n* = 6) (*p* < 0.001, Fig. 2A). This finding was further examined in a smaller independent dataset, GSE227550 (*n* = 3 tumor, *n* = 3 normal), which also showed a similar trend toward higher FOXP4 expression in tumor samples, although the difference did not reach statistical significance (*p* = 0.073, Fig. 2B).

To assess the relative position of FOXP4 within the context of differentially expressed genes across the entire transcriptome, a ranking analysis was performed on differentially expressed genes. As shown in the figure, FOXP4 is located within the region of significantly differentially expressed genes, with a positive log₂ fold change, indicating that it is consistently elevated in tumor tissues (Fig. 2C). This finding supports the potential involvement of FOXP4 in tumor-associated transcriptional regulatory networks.

To further investigate potential mechanisms underlying FOXP4 upregulation in colorectal cancer, we analyzed genomic alterations using TCGA datasets. Copy number variation analysis revealed that FOXP4 expression was significantly associated with genomic amplification, with higher expression observed in amplification groups compared with diploid samples (Fig. S1A). These results suggest that increased gene dosage may contribute, at least in part, to FOXP4 overexpression in CRC.

To determine the cellular origin of this elevated FOXP4 expression within the complex tumor microenvironment, we analyzed a publicly available single-cell RNA-sequencing (scRNA-seq) dataset from CRC patients (GSE166555). The UMAP visualization revealed distinct cell populations, including malignant epithelial cells, various immune cell types (e.g., T cells, B cells, plasma cells), and stromal cells (e.g., fibroblasts) (Fig. 2D, left panel). Critically, when we mapped FOXP4 expression onto these cell types, we observed a specific and high enrichment of FOXP4 transcripts predominantly within the malignant cell cluster, with minimal to no expression in the surrounding immune and stromal compartments (Fig. 2D, right panel). This single-cell resolution analysis strongly indicates that the observed upregulation of FOXP4 in bulk tumor tissue is driven primarily by the cancer cells themselves.

Next, to validate the protein expression level of FOXP4 in colorectal adenocarcinoma, we performed immunohistochemical staining in a clinical cohort encompassing diverse histological types and clinical stages, with a total of 38 cases analyzed. Overall, FOXP4 protein expression was upregulated in tumor tissues compared to adjacent normal tissues. It presents representative cases of FOXP4 overexpression across different histological subtypes of colorectal adenocarcinoma, including tubular adenocarcinoma (Cases 1–3), mucinous adenocarcinoma(Case 4), signet-ring cell carcinoma (Case 5) and adenosquamous carcinoma (Case 6) (Fig. 2E). Further T-test analysis of FOXP4 expression H-scores between adjacent normal tissues and tumor tissues showed statistical significance (Fig. 2F).

Finally, to investigate the clinical relevance of FOXP4 overexpression, we performed a survival analysis using a large, integrated patient cohort (*n* = 1167). A Kaplan-Meier analysis demonstrated that patients with high FOXP4 expression had a significantly shorter overall survival (OS) compared to those with low FOXP4 expression (Hazard Ratio [HR] = 1.68, 95% CI: 1.34–2.11; log-rank *p* = 5.9e-06, Fig. 2G).

Taken together, these results demonstrate that FOXP4 is consistently upregulated at both mRNA and protein levels in CRC, is predominantly expressed in malignant epithelial cells, and is associated with poor patient outcomes. Genomic analysis further suggests that FOXP4 overexpression may be partially driven by copy number amplification.

### Silencing of FOXP4 impairs proliferation, malignant potential, and tumorigenesis of CRC cells in vitro and in vivo

**Fig. 3:**
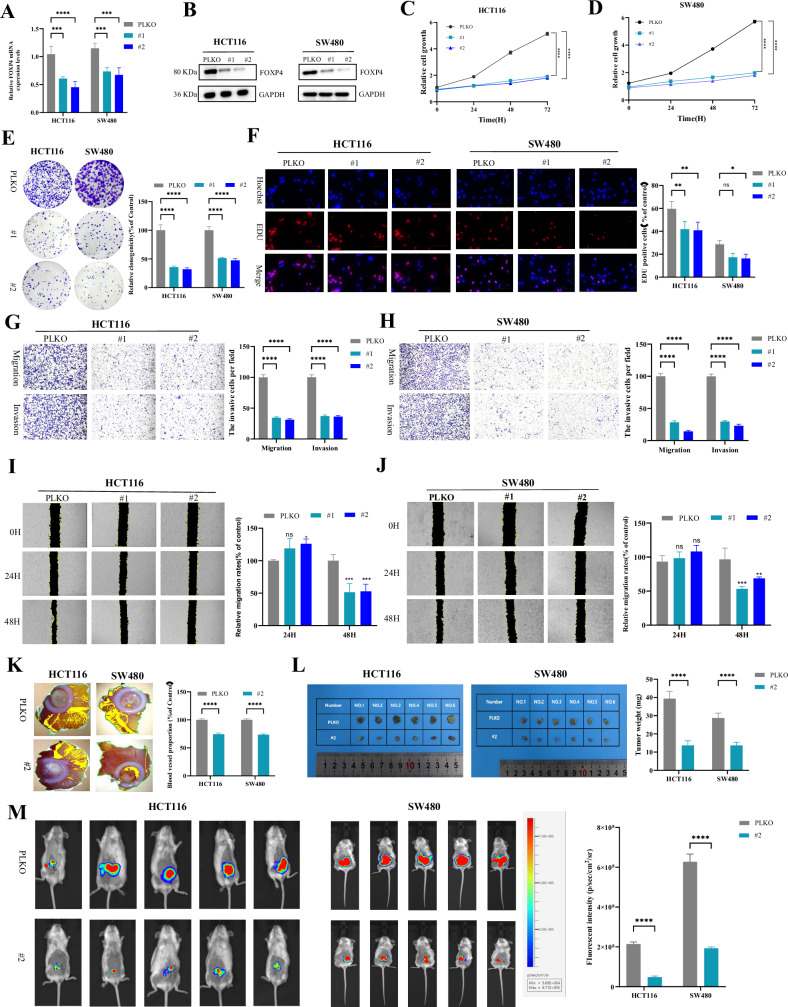
Silencing of FOXP4 impairs proliferation, malignant potential, and tumorigenesis of CRC cells in vitro and in vivo. Validation of FOXP4 knockdown efficiency in HCT116 and SW480 cells stably transfected with non-targeting control shRNA (PLKO) or two independent FOXP4-specific shRNAs (#1, #2). Efficiency was confirmed by qRT-PCR for mRNA levels (**A**) and Western blotting for protein levels (**B**). GAPDH served as a loading control. Cell growth curves for HCT116 (**C**) and SW480 (**D**) cells following FOXP4 knockdown, as measured by cell counting at 24, 48, and 72 h. **E** Colony formation assays of FOXP4-knockdown and control HCT116 and SW480 cells. Representative images are shown (left), and quantification of the relative number of colonies is presented as a bar graph (right). **F** EdU incorporation assays to assess cell proliferation. Representative immunofluorescence images (left) show nuclei (Hoechst, blue) and proliferating cells (EdU, red). Quantification of the percentage of EdU-positive cells is shown on the right. Transwell migration and invasion assays for HCT116 (**G**) and SW480 (**H**) cells with or without FOXP4 knockdown. Representative images of migrated/invaded cells are shown (left), with quantification of the average number of cells per field on the right. Wound-healing assays for HCT116 (**I**) and SW480 (**J**) cells. Representative images were taken at 0, 24, and 48 h after creating the scratch. The bar graphs show the quantification of the relative wound closure rate. In vivo chick chorioallantoic membrane (CAM) tumor growth assay. Stable HCT116 and SW480 cells (PLKO control vs. shFOXP4 #2) were inoculated onto CAMs. **K** Representative images of tumors on CAMs (left) and quantification of the blood vessel proportion surrounding the tumors (right). **L** Images of excised tumors (left) and quantification of tumor weight (right). **M** Murine orthotopic. Stable HCT116 and SW480 cells (PLKO control vs. shFOXP4 #2) were injected into the colons of mice. Representative in vivo bioluminescence images of nude mice bearing orthotopic tumors derived from HCT116 and SW480 cells stably expressing control vector (PLKO) or FOXP4 knockdown (#2). All quantitative data are presented as mean ± SD from at least three independent experiments. Statistical significance was determined by a one-way ANOVA followed by Tukey’s post-hoc test or a two-tailed Student’s *t* test where appropriate. *p < 0.05, **p < 0.01, ***p < 0.001, ****p < 0.0001; ns, not significant.

Our clinical data indicated that elevated FOXP4 expression is a hallmark of aggressive CRC. To directly test the hypothesis that FOXP4 functions as an oncogenic driver, we performed loss-of-function experiments in two well-established CRC cell lines, HCT116 and SW480, which exhibit relatively high endogenous levels of FOXP4. We stably knocked down FOXP4 expression using two independent short hairpin RNAs (shRNA #1 and shRNA #2), with a non-targeting shRNA (PLKO) serving as the control. The knockdown efficiency was robustly validated at both the mRNA and protein levels. Quantitative real-time PCR (qRT-PCR) and Western blot analyses confirmed a significant reduction of FOXP4 in both HCT116 and SW480 cells treated with either shRNA construct compared to the PLKO control (Fig. 3A, B).

We first assessed the impact of FOXP4 depletion on cell proliferation. Cell counting assays revealed that FOXP4 knockdown significantly suppressed the growth rate of both HCT116 and SW480 cells over a 72-hour period (Fig. 3C, D). This anti-proliferative effect was further substantiated by colony formation assays, which measure long-term proliferative capacity. The ability of both cell lines to form colonies was markedly diminished following FOXP4 knockdown, as evidenced by a drastic reduction in both the number and size of colonies compared to the control group (Fig. 3E). To further quantify cell proliferation at a single-cell level, we performed an EdU (5-ethynyl-2’-deoxyuridine) incorporation assay. The percentage of EdU-positive cells, representing cells undergoing active DNA synthesis, was significantly decreased in the FOXP4-silenced HCT116 and SW480 populations (Fig. 3F). Collectively, these results demonstrate that FOXP4 is essential for maintaining the proliferative capacity of CRC cells.

Next, we investigated the role of FOXP4 in regulating the migratory and invasive capabilities of CRC cells, which are key hallmarks of metastasis. Using Transwell assays, we found that FOXP4 knockdown dramatically impaired both the migration (through an uncoated membrane) and invasion (through a Matrigel-coated membrane) of HCT116 and SW480 cells (Fig. 3G, H). This was complemented by wound-healing assays, which showed that the closure of a scratch wound was significantly delayed at 48 h in FOXP4-depleted cells compared to the rapid closure observed in control cells, confirming a defect in collective cell migration (Fig. 3I, J).

To extend these in vitro findings to an in vivo context, we employed the chick chorioallantoic membrane (CAM) assay and an orthotopic colorectal cancer model, two well-established models to assess tumor growth and angiogenesis. HCT116 and SW480 cells stably expressing control shRNA or FOXP4 shRNA #2 were grafted onto the CAMs of chicken embryos and injected into the colons of mice. After 8 days, tumors formed by control cells were visibly larger and displayed a dense, spoke-wheel pattern of blood vessels converging on the tumor mass, indicative of robust angiogenesis. In stark contrast, tumors formed by FOXP4-knockdown cells were significantly smaller and exhibited a substantially reduced blood vessel supply (Fig. 3K). The macroscopic tumors were then excised and weighed. Consistent with the visual observations, the average weight of tumors derived from FOXP4-depleted cells was significantly lower than that of the control tumors for both HCT116 and SW480 cell lines (Fig. 3L). Tumor progression was monitored longitudinally using bioluminescence imaging at Day 8 post-injection. FOXP4 knockdown resulted in a significant reduction in bioluminescence intensity compared to the control group, indicating impaired tumor growth (Fig. 3M).

In summary, these comprehensive functional assays, both in vitro and in vivo, provide compelling evidence that FOXP4 plays a critical role in promoting multiple hallmarks of cancer in CRC, including proliferation, clonogenicity, migration, invasion, and angiogenesis-dependent tumor growth.

### FOXP4 directly binds to the bag3 promoter and transcriptionally activates its expression

**Fig. 4:**
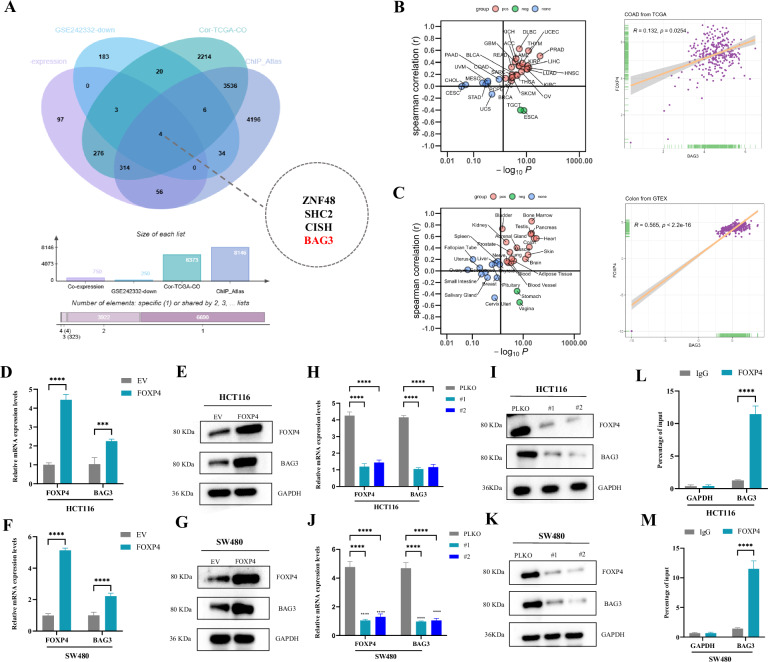
FOXP4 directly binds to the BAG3 promoter and transcriptionally activates its expression. **A** Venn diagram illustrating the bioinformatic screening strategy to identify direct downstream targets of FOXP4. The intersection of four datasets—genes downregulated upon FOXP4 knockdown (GSE242332), genes co-expressed with FOXP4 (COXPRESdb and TCGA-COAD), and predicted targets from ChIP-Atlas—yielded four high-confidence candidates, including BAG3. Correlation analysis of FOXP4 and BAG3 mRNA expression. Scatter plots show a significant positive correlation in the TCGA-COAD tumor cohort (*n* = 473) (**B**) and a strong positive correlation in normal colon tissue from the GTEx database (*n* = 389) (**C**). Spearman’s correlation coefficient (R) and p-value are indicated. Ectopic expression of FOXP4 induces BAG3 expression. HCT116 (**D, E**) and SW480 (**F, G**) cells were transfected with an empty vector (EV) or a FOXP4-expressing vector. The mRNA levels of FOXP4 and BAG3 were measured by qRT-PCR (**D, F**), and the protein levels were assessed by Western blotting (**E, G**). Silencing of FOXP4 reduces BAG3 expression. HCT116 (**H, I**) and SW480 (**J, K**) cells were stably transduced with control (PLKO) or two independent FOXP4-specific shRNAs. The mRNA levels of FOXP4 and BAG3 were measured by qRT-PCR (**H, J**), and the protein levels were assessed by Western blotting (**I, K**). GAPDH was used as a loading control. Chromatin Immunoprecipitation (ChIP)-qPCR analysis in HCT116 (**L**) and SW480 (**M**) cells. Endogenous FOXP4 was immunoprecipitated, and the enrichment of the BAG3 promoter region was quantified by qPCR. Immunoprecipitation with a non-specific IgG served as a negative control, and the GAPDH promoter served as a negative locus control. Data are presented as a percentage of input. All quantitative data are presented as mean ± SD from three independent experiments. Statistical significance was determined by a two-tailed Student’s *t* test or one-way ANOVA. ***p < 0.001, ****p < 0.0001.

Having established FOXP4 as a critical pro-tumorigenic factor in CRC, we next sought to elucidate the downstream molecular mechanism by which it exerts its function. As a transcription factor, FOXP4 likely regulates a specific set of target genes. To identify these candidate targets, we adopted a multi-pronged strategy integrating several independent datasets (Fig. 4A). First, we identified genes downregulated upon FOXP4 silencing in the CRC dataset GSE242332. Second, we collected genes positively co-expressed with FOXP4 in the TCGA-COAD cohort. Third, we used ChIP-Atlas to predict potential FOXP4 binding targets. Finally, we included the top FOXP4 co-expressed genes from COXPRESdb. The intersection of these four datasets yielded four candidate downstream genes: ZNF48, SHC2, CISH, and BAG3 (Fig. 4A). Among these, BCL2-associated athanogene 3 (BAG3), a known anti-apoptotic and pro-survival protein, drew our attention due to its emerging roles in cancer progression and therapy resistance.

To initially explore the relationship between FOXP4 and BAG3, we analyzed their expression correlation in publicly available patient-derived and normal tissue cohorts. In the TCGA-COAD dataset, FOXP4 and BAG3 mRNA levels showed a modest but statistically significant positive correlation (R = 0.132, *p* = 0.0254) in tumor tissues (Fig. 4B). Notably, a stronger positive correlation was observed in normal colon tissues from the GTEx database (R = 0.565, *p* < 2.2× 10^−16^) (Fig. 4C). Given that the correlation in tumor tissues was relatively modest, the functional relationship between FOXP4 and BAG3 was further investigated experimentally.

To experimentally validate whether FOXP4 regulates BAG3 expression, we first performed gain-of-function experiments. We transiently overexpressed FOXP4 in HCT116 and SW480 cells, using an empty vector (EV) as a control. As confirmed by qRT-PCR and Western blot, the overexpression of FOXP4 led to an upregulation of BAG3 at both the mRNA and protein levels in both cell lines (Fig. 4D–G). Conversely, we performed loss-of-function experiments by knocking down FOXP4 using two independent shRNAs. The stable silencing of FOXP4 resulted in a downregulation of BAG3 mRNA and protein expression in both HCT116 and SW480 cells (Fig. 4H–K). These complementary experiments provide evidence of a positive regulatory relationship between FOXP4 and BAG3 in CRC cells.

To determine whether BAG3 represents a direct transcriptional target of FOXP4 rather than a secondary downstream effector, we performed Chromatin Immunoprecipitation (ChIP)-qPCR. Analysis of the BAG3 promoter region revealed significant enrichment of FOXP4 occupancy compared to IgG controls (Fig. 4L, M), indicating that FOXP4 directly binds the BAG3 promoter and contributes to BAG3 transcriptional regulation. Taken together, these results support BAG3 as a direct transcriptional target of FOXP4 in colorectal cancer cells.

### The FOXP4-BAG3 axis is associated with the emt-related transcriptional programs

**Fig. 5:**
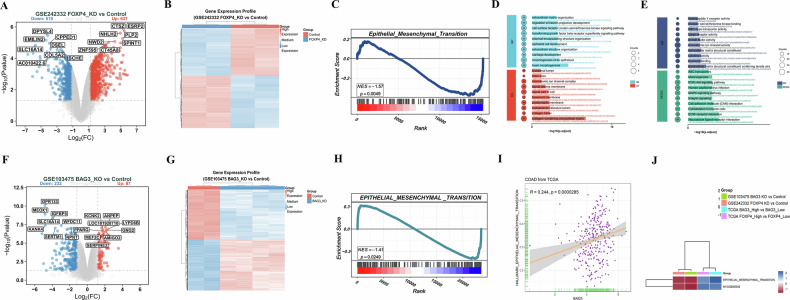
The FOXP4-BAG3 axis is associated with the EMT-related transcriptional programs. Transcriptomic analysis of the GSE242332 dataset comparing FOXP4 knockdown (FOXP4_KD) cells to control cells. Volcano plot (**A**) shows 637 upregulated and 519 downregulated genes. Heatmap (**B**) displays the expression profile of these differentially expressed genes. **C** Gene Set Enrichment Analysis (GSEA) plot showing a significant negative enrichment of the Hallmark EMT gene set in FOXP4_KD cells versus control cells (NES = −1.57). Functional enrichment analysis of differentially expressed genes in the FOXP4_KD dataset. Bar plots show the top enriched Gene Ontology (GO) terms for Biological Process (BP) and Cellular Component (CC) (**D**), Molecular Function (MF) and top enriched Kyoto Encyclopedia of Genes and Genomes (KEGG) pathways (**E**). Transcriptomic analysis of the GSE103475 dataset comparing BAG3 knockout (BAG3_KO) cells to control cells. Volcano plot (**F**) shows 87 upregulated and 232 downregulated genes. Heatmap (**G**) displays their expression profile. **H** GSEA plot showing a significant negative enrichment of the Hallmark EMT gene set in BAG3_KO cells versus control cells (NES = −1.41), mirroring the result from the FOXP4_KD dataset. **I** Correlation analysis in the TCGA-COAD cohort. The scatter plot shows a significant positive correlation between BAG3 mRNA expression and the enrichment score of the Hallmark EMT gene set (R = 0.244, *p* < 0.0001). **J** Clustered heatmap summarizing the activity of the EMT pathway across different datasets. The analysis shows that high expression of FOXP4 or BAG3 (in TCGA) is associated with higher EMT scores, while knockdown/knockout of FOXP4 or BAG3 is associated with lower EMT scores.

Having established that FOXP4 directly regulates BAG3, we next aimed to identify the downstream cellular programs controlled by this regulatory axis. To this end, we first analyzed a publicly available transcriptomic dataset (GSE242332) from cells with FOXP4 knockdown (FOXP4_KD). Differential expression analysis revealed a significant alteration in the gene expression profile, with 637 genes being upregulated and 519 genes downregulated upon FOXP4 depletion (Fig. 5A, B).

To uncover the biological functions associated with FOXP4, we performed Gene Set Enrichment Analysis (GSEA) on this dataset. Strikingly, GSEA revealed a significant negative enrichment for the “Hallmark_Epithelial_Mesenchymal_Transition” gene set in the FOXP4_KD group compared to the control group (NES = −1.57, *p* = 0.0049) (Fig. 5C). This finding suggests that high levels of FOXP4 are positively associated with the activation of the EMT program, a key process for cancer invasion and metastasis. Consistently, analysis of TCGA colorectal cancer datasets showed that FOXP4 expression was positively correlated with EMT-related transcriptional signatures (Spearman r = 0.23, *p* = 1 × 10⁻⁴) (Fig. S1B), further supporting the association between FOXP4 and EMT-related programs in clinical samples. To further explore this, we conducted Gene Ontology (GO) and Kyoto Encyclopedia of Genes and Genomes (KEGG) pathway analyses on the differentially expressed genes. The downregulated genes upon FOXP4 knockdown were significantly enriched in biological processes (BP) such as “extracellular structure organization,” “cell-cell adhesion,” and “epithelial cell proliferation,” and cellular components (CC) like “collagen-containing extracellular matrix” and “integrin complex” (Fig. 5D). Conversely, upregulated genes were enriched in molecular functions (MF) such as “receptor ligand activity” and “cytokine activity”. KEGG analysis of the downregulated genes highlighted pathways including “ECM-receptor interaction,” “PI3K-Akt signaling pathway,” “Focal adhesion,” and “MAPK signaling pathway,” all of which are intimately linked to EMT and cell motility (Fig. 5E).

Given that BAG3 is a direct target of FOXP4, we hypothesized that BAG3 might mediate this effect on the EMT program. To test this, we analyzed another independent transcriptomic dataset (GSE103475) from cells with BAG3 knockout (BAG3_KO). Consistent with the FOXP4_KD data, BAG3_KO also led to a significant perturbation of the transcriptome, with 87 genes upregulated and 232 genes downregulated (Fig. 5F, G). Remarkably, a GSEA performed on this BAG3_KO dataset mirrored our previous finding: the “Hallmark_Epithelial_Mesenchymal_Transition” gene set was also significantly negatively enriched in the BAG3_KO group (NES = −1.41, *p* = 0.0249) (Fig. 5H). This parallel result suggests that both FOXP4 and its target BAG3 contribute to EMT-associated transcriptional programs.

To extend this association to clinical samples, we analyzed the correlation between BAG3 expression and the EMT hallmark gene set score in the TCGA-COAD patient cohort. This analysis revealed a significant positive correlation (R = 0.244, *p* < 0.0001), indicating that tumors with higher BAG3 levels tend to have a more active EMT signature (Fig. 5I). Finally, a clustered heatmap analysis integrating data from the FOXP4_KD, BAG3_KO, and TCGA cohorts demonstrated a consistent pattern: conditions with high FOXP4 or high BAG3 expression were associated with higher activity of the EMT pathway, while conditions with low FOXP4 or BAG3 were associated with lower EMT activity (Fig. 5J).

Collectively, these analyses support an association between the FOXP4–BAG3 axis and EMT-related transcriptional programs, providing a mechanistic framework for the pro-invasive phenotypes observed in our functional assays.

### Restoration of BAG3 expression partially rescues the malignant phenotypes suppressed by FOXP4 knockdown

Our bioinformatic analyses strongly indicated that the FOXP4-BAG3 axis promotes the EMT program. To experimentally validate this and to determine whether BAG3 is the key functional mediator of FOXP4’s oncogenic effects, we performed a series of rescue experiments. We first stably knocked down FOXP4 in HCT116 and SW480 cells (group “#2”) and then, in these FOXP4-depleted cells, ectopically re-expressed a BAG3 construct that is resistant to shRNA-mediated degradation (group “#2 + BAG3”). Control cells were transduced with an empty vector (PLKO).

**Fig. 6:**
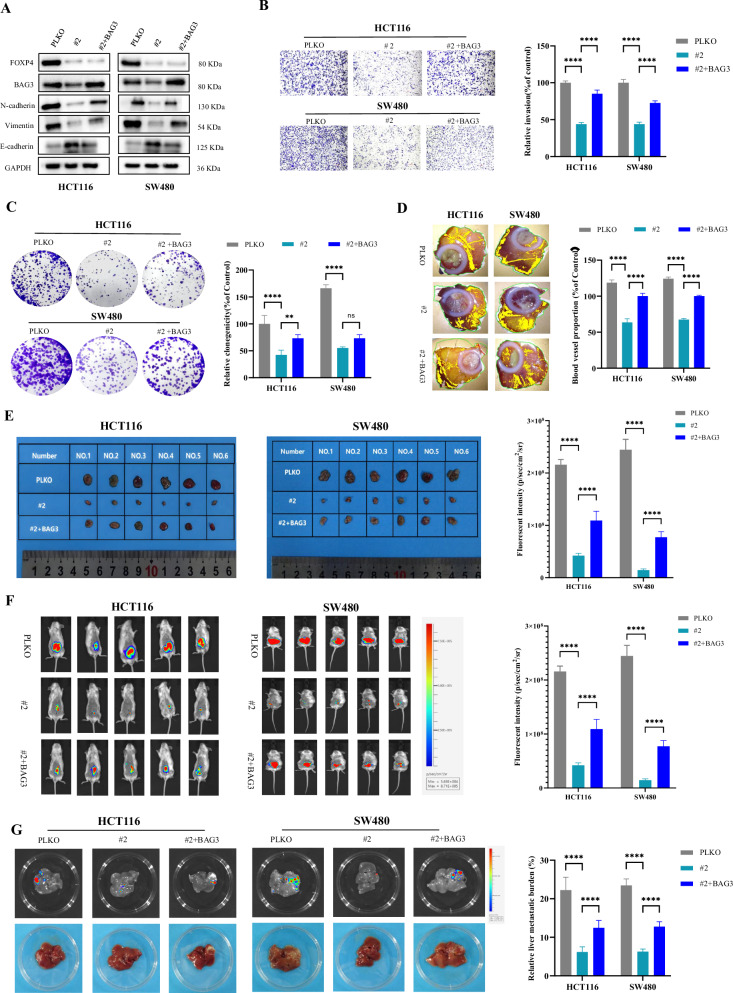
Restoration of BAG3 expression partially rescues the malignant phenotypes suppressed by FOXP4 knockdown. Rescue experiments were performed in HCT116 and SW480 cells with three groups: control (PLKO), FOXP4 knockdown (shFOXP4 #2), and FOXP4 knockdown with concurrent BAG3 re-expression (#2 + BAG3). **A** Western blot analysis of FOXP4, BAG3, and key EMT markers (E-cadherin, N-cadherin, Vimentin) in the three experimental groups for both HCT116 and SW480 cells. GAPDH served as the loading control. **B** Transwell invasion assay. Representative images of invaded cells (left) and quantification of relative invasion (right) for the three groups. FOXP4 knockdown inhibits invasion, while BAG3 re-expression restores it. **C** Colony formation assay. Representative images of colonies (left) and quantification of relative clonogenicity (right). The suppressed colony-forming ability due to FOXP4 silencing is significantly rescued by BAG3 re-expression. **D** In vivo CAM tumor angiogenesis assay. Representative images of tumors on CAMs with color-coded blood vessel analysis (left) and quantification of the blood vessel proportion (right). BAG3 re-expression restores the pro-angiogenic effect that was lost upon FOXP4 knockdown. **E** In vivo CAM tumor growth assay. Representative images of excised tumors from the three groups (left) and quantification of tumor weight (right). The reduction in tumor mass caused by FOXP4 knockdown is significantly reversed by restoring BAG3 expression. **F** Murine orthotopic. Representative in vivo bioluminescence images of nude mice bearing orthotopic tumors derived from HCT116 and SW480 cells stably expressing control vector (PLKO), FOXP4 knockdown (#2), and FOXP4 knockdown (#2 + BAG3) (*n* = 5). **G** Ex vivo bioluminescence imaging and gross morphology of excised livers from the intrasplenic injection liver metastasis model, with quantification of liver metastatic burden. The upper panel shows representative ex vivo bioluminescence images of excised livers, and the lower panel shows the corresponding gross liver images. The bar graph on the right shows quantitative analysis of liver metastatic burden, expressed as Tumor area/Liver area (%) (*n* = 5). All quantitative data are presented as mean ± SD from at least three independent experiments. Statistical significance was determined by a one-way ANOVA followed by Tukey’s post-hoc test. **p < 0.01, ****p < 0.0001; ns, not significant.

First, we examined the expression of key EMT markers by Western blot. As expected, knockdown of FOXP4 (group “#2”) led to a significant downregulation of its target, BAG3. Concurrently, FOXP4 silencing induced a classic mesenchymal-to-epithelial transition (MET) phenotype, characterized by a marked increase in the epithelial marker E-cadherin and a concomitant decrease in the mesenchymal markers N-cadherin and Vimentin. Crucially, re-expression of BAG3 in these FOXP4-knockdown cells (group “#2 + BAG3”) substantially reversed this effect. While FOXP4 levels remained low, BAG3 expression was restored, leading to the downregulation of E-cadherin and the upregulation of N-cadherin and Vimentin, effectively rescuing the EMT molecular signature (Fig. 6A).

Next, we assessed whether this molecular rescue translated to a functional rescue of the malignant phenotypes. In Transwell invasion assays, FOXP4 knockdown drastically reduced the invasive capacity of both HCT116 and SW480 cells. Remarkably, restoring BAG3 expression in these cells significantly recovered their invasive potential, bringing it back to levels approaching that of the control group (Fig. 6B). Similarly, the profound suppression of long-term proliferation caused by FOXP4 silencing, as measured by colony formation assays, was also significantly rescued by the reintroduction of BAG3. The number and size of colonies formed by the "#2 + BAG3" group were substantially greater than those in the FOXP4-knockdown group (Fig. 6C). It is noteworthy that in SW480 cells, the rescue of clonogenicity was nearly complete, whereas in HCT116 cells, the rescue was significant but partial, suggesting potential cell line-specific dependencies or the existence of minor, BAG3-independent pathways downstream of FOXP4 in this cell line.

Finally, we tested the relevance of this axis in vivo using the CAM assay and an orthotopic colorectal cancer model. As previously observed, tumors formed by FOXP4-knockdown cells (group “#2”) were small and poorly vascularized. In striking contrast, tumors from the BAG3-rescue group (“#2 + BAG3”) were visibly larger and exhibited a restoration of the dense angiogenic network, comparable to the control group (Fig. 6D). Measurement of the excised tumor weights provided quantitative confirmation of this observation. The significant reduction in tumor weight caused by FOXP4 depletion was almost completely reversed upon restoration of BAG3 expression in both HCT116 and SW480 cell-derived tumors (Fig. 6E). Similarly, tumor progression was monitored longitudinally by bioluminescence imaging on day 8 after implantation. Compared to the control group (PLKO), tumors formed by FOXP4-knockdown cells (#2) exhibited minimal bioluminescence intensity. In stark contrast, the BAG3-restored group (“#2 + BAG3”) demonstrated significantly greater bioluminescence intensity (Fig. 6F).

To further assess the role of this axis in metastatic progression, we established an in vivo liver metastasis model by intrasplenic injection. FOXP4 knockdown markedly reduced liver metastatic burden, as indicated by lower ex vivo bioluminescence signals and fewer visible metastatic nodules on the liver surface. BAG3 restoration partially rescued this phenotype, leading to increased liver metastatic burden compared with the FOXP4-knockdown group (Fig. 6G). H&E staining further confirmed these findings, showing fewer and smaller metastatic lesions in the FOXP4-knockdown group and partial restoration of metastatic colonization in the BAG3-rescue group (Fig. S2). Together, these results suggest that FOXP4 promotes CRC cell invasion, proliferation, tumor growth, angiogenesis, and liver metastatic colonization, at least in part through direct transcriptional activation of BAG3.

### Silencing of BAG3 attenuates the oncogenic phenotypes induced by FOXP4 overexpression

To further examine whether FOXP4 exerts its oncogenic functions, at least in part, through BAG3, we performed the reciprocal rescue experiment. We ectopically overexpressed FOXP4 in HCT116 and SW480 cells (group "FOXP4"), which have relatively low basal expression, and in parallel, co-expressed FOXP4 with an shRNA targeting BAG3 (group "FOXP4+shBAG3"). Cells transfected with an empty vector (EV) served as the control.

**Fig. 7:**
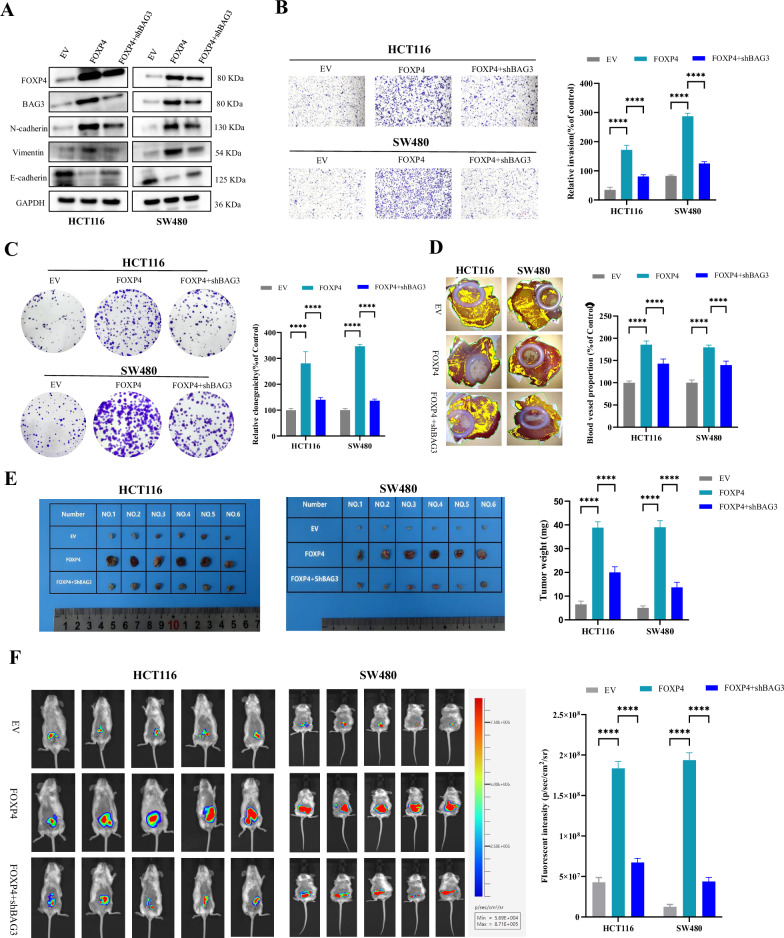
Silencing of BAG3 attenuates the oncogenic phenotypes induced by FOXP4 overexpression. Rescue experiments were performed in HCT116 and SW480 cells with three groups: control (Empty Vector, EV), FOXP4 overexpression (FOXP4), and FOXP4 overexpression with concurrent BAG3 knockdown (FOXP4+shBAG3). **A** Western blot analysis of FOXP4, BAG3, and EMT markers (N-cadherin, Vimentin, E-cadherin) in the three experimental groups for both HCT116 and SW480 cells. GAPDH was used as a loading control. **B** Transwell invasion assay. Representative images of invaded cells (left) and quantification of relative invasion (right). The enhanced invasion caused by FOXP4 overexpression is significantly attenuated by BAG3 silencing. **C** Colony formation assay. Representative images of colonies (left) and quantification of relative clonogenicity (right). The increased colony-forming ability due to FOXP4 overexpression is abrogated by concurrent BAG3 knockdown. **D** In vivo CAM tumor angiogenesis assay. Representative images of tumors on CAMs with color-coded blood vessel analysis (left) and quantification of the blood vessel proportion (right). BAG3 knockdown blunts the pro-angiogenic effect induced by FOXP4 overexpression. **E** In vivo CAM tumor growth assay. Images of excised tumors from the three groups (left) and quantification of tumor weight (right). The increased tumor mass driven by FOXP4 overexpression is significantly reversed by silencing BAG3. **F** Murine orthotopic. Representative bioluminescence images of nude mice bearing orthotopic tumors derived from HCT116 and SW480 cells stably expressing control vector (EV), FOXP4 overexpression (FOXP4), and FOXP4 overexpression with concurrent BAG3 knockdown (FOXP4+shBAG3) (*n* = 5). All quantitative data are presented as mean ± SD from at least three independent experiments. Statistical significance was determined by a one-way ANOVA followed by Tukey’s post-hoc test. ****p < 0.0001.

First, we validated the expression of the relevant proteins by Western blot. As intended, cells in the "FOXP4" group showed a marked increase in both FOXP4 and its downstream target, BAG3. In the "FOXP4+shBAG3" group, FOXP4 remained overexpressed, but the co-transfection of shBAG3 successfully prevented the corresponding increase in BAG3 protein levels, bringing them down to a level even lower than the EV control. We then assessed the impact on EMT markers. Overexpression of FOXP4 alone induced an EMT-like phenotype, characterized by the downregulation of E-cadherin and upregulation of N-cadherin and Vimentin. Strikingly, this molecular shift was almost completely reversed in the "FOXP4+shBAG3" group, where E-cadherin expression was restored, and N-cadherin and Vimentin levels were reduced, despite the continued high expression of FOXP4 (Fig. 7A).

Consistent with these EMT marker changes, modulation of BAG3 expression was accompanied by corresponding changes in β-catenin and ZEB1 levels (Fig. S3A), suggesting that BAG3 may influence key downstream effectors of EMT-associated signaling. To further examine whether BAG3 contributes to the stability of EMT-associated proteins, we performed cycloheximide (CHX) chase assays. BAG3 knockdown accelerated the decline of ZEB1 protein levels over time (Fig. S3B), suggesting that BAG3 contributes to the stability of EMT-associated effectors.

We next determined if this molecular reversal translated to a functional blockade. Overexpression of FOXP4 significantly enhanced the invasive capabilities of both HCT116 and SW480 cells in Transwell assays. However, this pro-invasive effect was significantly attenuated when BAG3 was simultaneously knocked down. The number of invading cells in the "FOXP4+shBAG3” group was dramatically lower than in the "FOXP4” group and was statistically comparable to the basal invasion of the control cells (Fig. 7B). A similar pattern was observed in the colony formation assays. While FOXP4 overexpression robustly promoted the clonogenic growth of both cell lines, this effect was largely abrogated by the concurrent silencing of BAG3. The number and size of colonies in the "FOXP4+shBAG3“ group were significantly reduced compared to the FOXP4-overexpressing group (Fig. 7C).

Finally, we validated this critical dependency in vivo using the CAM model. Tumors formed by FOXP4-overexpressing cells were substantially larger and induced a more pronounced angiogenic response compared to control tumors. In stark contrast, tumors from the "FOXP4+shBAG3” group were markedly smaller, and the pro-angiogenic effect driven by FOXP4 was significantly blunted (Fig. 7D). Quantitative analysis of the excised tumor weights confirmed these observations. The dramatic increase in tumor mass driven by FOXP4 overexpression was significantly negated by the concurrent knockdown of BAG3 in both HCT116 and SW480-derived tumors (Fig. 7E). Similarly, tumor progression was monitored longitudinally using bioluminescence imaging at Day 8 post-injection. Compared to the control group (EV), tumors formed by FOXP4-overexpressing cells (“FOXP4”) exhibited significantly higher bioluminescence intensity. In stark contrast, the bioluminescence intensity in the BAG3-knockdown group (“FOXP4+shBAG3”) was markedly lower (Fig. 7F).

Collectively, these findings indicate that BAG3 acts as an important mediator of FOXP4-driven EMT-associated phenotypes and tumor progression.

### Single-cell transcriptomic analysis supports epithelial-specific relevance of the FOXP4–BAG3 axis

To further evaluate the clinical and epithelial-specific relevance of the FOXP4–BAG3 axis, we analyzed a publicly available colorectal cancer single-cell RNA-sequencing dataset (GSE166555). Within malignant epithelial cells, FOXP4 expression showed a significant positive correlation with BAG3 expression (Spearman r = 0.40, *p* < 4.6 × 10^−41^) (Fig. S4A). Moreover, malignant epithelial cells with high FOXP4 expression exhibited significantly elevated EMT module scores compared with FOXP4-low cells (Wilcoxon test, *p* = 0.0107) (Fig. S4B). These findings extend our observations from established CRC cell lines to patient-derived malignant epithelial cells at single-cell resolution and support an association between the FOXP4–BAG3 axis and EMT-related transcriptional programs in clinically relevant tumor epithelial cells.

**Fig. 8:**
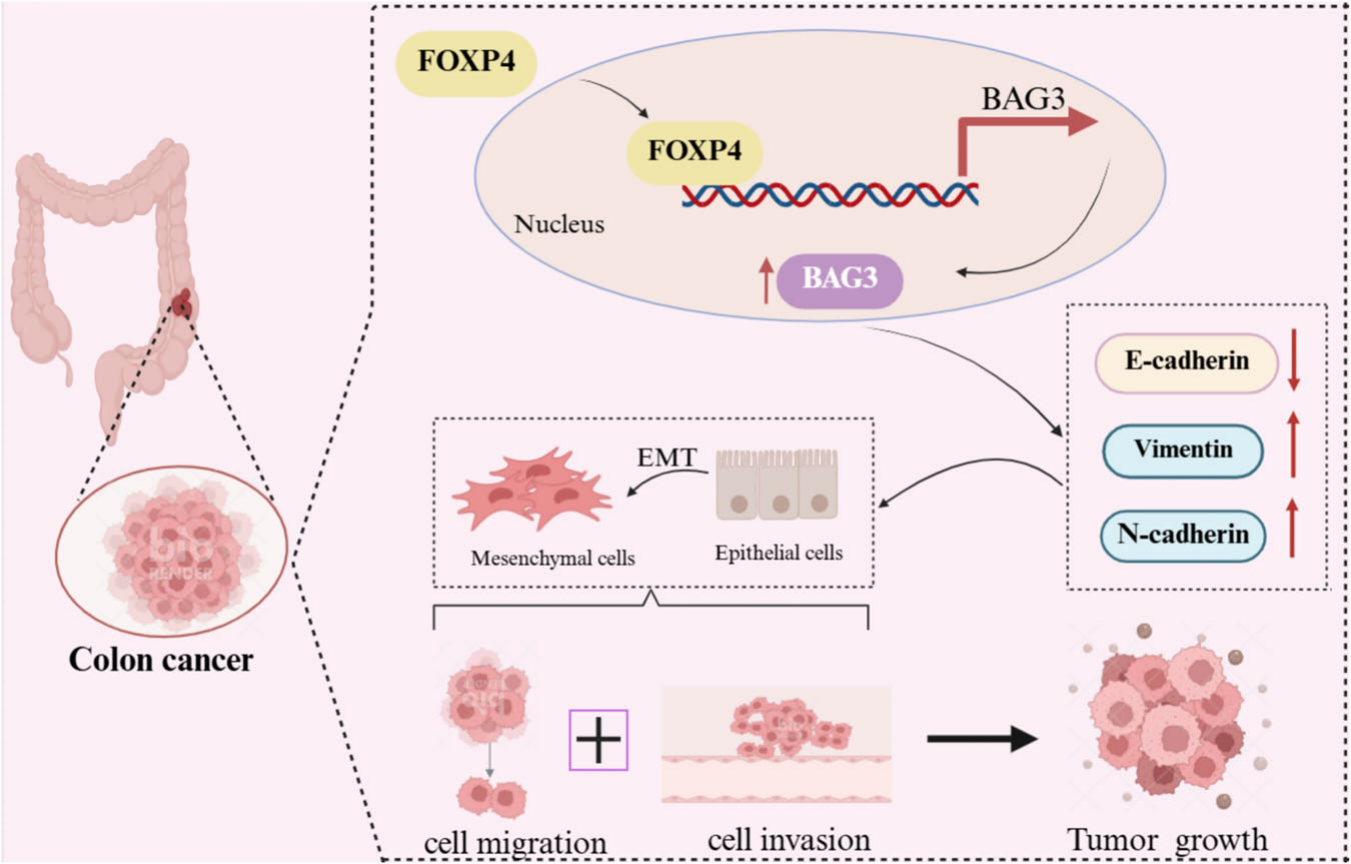
Schematic illustration of the FOXP4-BAG3 axis driving colorectal cancer progression. In colorectal cancer cells, FOXP4 binds to the promoter region of BAG3 and induces its expression. Increased BAG3 expression is associated with EMT-related molecular changes, including reduced E-cadherin and increased Vimentin and N-cadherin, thereby contributing to enhanced migratory, invasive, and tumor-promoting phenotypes.

Collectively, these experiments indicate that BAG3 is not only downstream of FOXP4 but also functionally important for mediating its pro-tumorigenic effects. The ability of FOXP4 to promote EMT-associated phenotypes, invasion, proliferation, and tumorigenesis appears to be largely dependent on its capacity to upregulate BAG3 expression (Fig. 8).

## Discussion

The metastatic progression of colorectal cancer (CRC) remains a formidable clinical challenge, driven by complex transcriptional networks that endow cancer cells with malignant plasticity. In this study, we identified a FOXP4–BAG3 axis that contributes to EMT-associated changes, invasive behavior, and metastatic progression in CRC. Our findings suggest that FOXP4 promotes these malignant features, at least in part, by directly activating BAG3.

Our investigation was initiated by an unbiased bioinformatic screen that pinpointed FOXP4 as a consistently and significantly upregulated transcription factor across multiple independent CRC patient cohorts. This observation was further corroborated by single-cell analysis, which revealed that the overexpression of FOXP4 is largely due to characteristics inherent to the tumor cells themselves, and clinically, its high expression serves as an independent predictor of poor patient survival. At the same time, FOXP4 is unlikely to act alone. Its association with copy-number gain and EMT-related transcriptional features suggests that it functions within a broader oncogenic network rather than as a single determinant of tumor progression.

A key finding of this study is that BAG3 serves as a direct downstream target of FOXP4. Integrative screening and experimental validation showed that FOXP4 binds to the BAG3 promoter and activates its transcription, thereby linking FOXP4-dependent transcriptional regulation to BAG3-associated stress adaptation programs. Although the correlation between FOXP4 and BAG3 was modest in large tumor datasets, this is likely influenced by tissue heterogeneity. More importantly, their biological connection is supported by promoter binding, transcriptional regulation, and rescue experiments.

Functional studies further showed that BAG3 mediates a substantial part of the FOXP4-dependent malignant phenotype. Transcriptomic analyses indicated that both FOXP4 and BAG3 are associated with epithelial–mesenchymal transition (EMT)-related processes, consistent with the observed changes in EMT markers and invasive behavior. BAG3 re-expression partially restored the phenotypes suppressed by FOXP4 knockdown, whereas BAG3 silencing attenuated the effects induced by FOXP4 overexpression. Together, these findings place BAG3 downstream of FOXP4 and support its role as an important effector of FOXP4-induced malignant plasticity, while suggesting promotion of EMT-associated phenotypes rather than complete control of the EMT program.

The downstream signaling mechanisms require further clarification. In our system, BAG3 modulation was accompanied by corresponding changes in β-catenin and ZEB1. In addition, an exploratory CHX chase assay showed that ZEB1 protein declined more rapidly after BAG3 knockdown, suggesting that BAG3 may contribute to the maintenance of EMT-related protein stability. These findings indicate that BAG3 may help transmit FOXP4-dependent transcriptional output to pathways associated with epithelial plasticity and invasion. At present, this should be regarded as a mechanistic clue rather than a fully defined signaling hierarchy, and whether β-catenin, ZEB1, or related pathways are directly regulated by BAG3 in this context requires further investigation.

The in vivo data further support the biological relevance of these findings. FOXP4 depletion reduced the burden of liver metastases in a liver metastasis model, suggesting that the role of this axis is not limited to cell culture systems. This is particularly important because it supports the relevance of the FOXP4–BAG3 pathway in metastatic progression under more physiologically relevant conditions.

Several limitations should be acknowledged. Some of the public validation datasets used in this study were relatively small, which may reduce statistical power and limit generalizability. Therefore, further validation in larger, independent cohorts would strengthen the robustness of these findings. Furthermore, although the cell line and in vivo models used in this study support the biological relevance of the FOXP4–BAG3 axis, they may not fully capture patient-specific epithelial states, genetic heterogeneity, or the full extent of epithelial plasticity observed in clinical tumors. While our additional single-cell transcriptomic analysis extends these findings to patient-derived malignant epithelial cells, functional validation in primary patient-derived systems will be required to further strengthen the translational relevance of this axis. Finally, BAG3 is unlikely to be the only relevant downstream effector of FOXP4 in colorectal cancer. Broader mapping approaches, including ChIP-seq together with more extensive functional studies, will be required to more comprehensively define the FOXP4 regulatory network and to determine whether this axis could have therapeutic potential.

In summary, this study identified a FOXP4–BAG3 regulatory axis associated with EMT-related invasion and metastatic progression in colorectal cancer. By demonstrating that BAG3 is a direct transcriptional target of FOXP4 and a functional mediator of its malignant effects, our study provides additional insight into the mechanisms of epithelial plasticity in colorectal cancer and supports further investigation of this pathway in biologically and clinically relevant settings.

## Supplementary information

Supplementary Figure Information

Reproducibility checklist

Original Western Blot image

## Data Availability

The datasets analyzed in this study are publicly available from the Gene Expression Omnibus (GEO), The Cancer Genome Atlas (TCGA), and other publicly accessible online resources as described in the Materials and Methods. Additional information is available from the corresponding author upon reasonable request.

## Code availability

Custom R scripts used for data processing, bioinformatic analysis, and figure generation in this study are available from the corresponding author upon reasonable request.
