# Supplementary material for: FOXP4 promotes metastatic progression in colorectal cancer through transcriptional activation of BAG3
Source: Cell Death Dis. 2026 Jun 11;17(1):739. doi: 10.1038/s41419-026-08941-5 (PMC13478455; doi:10.1038/s41419-026-08941-5)
Supplement: Supplementary file 1 [file 41419_2026_8941_MOESM2_ESM.pdf]

Corresponding Author Name: Bin Liu
Manuscript Number: CDDIS-26-016

SPRINGER NATURE

Reporting Summary

Springer Nature wishes to improve the reproducibility of the work that we publish. This checklist is used to ensure good reporting standards and to improve the reproducibility. Please respond completely to all questions relevant to your manuscript. For more information, please read the journal's Guide to Authors.

☑ **Check here to confirm that the following information is available in the Material & Methods section:**

- **The exact sample size (*n*)** for each experimental group/condition, given as a number, not a range
- **A description of the sample collection** allowing the reader to understand whether the samples represent technical or biological replicates (including how many animals, litters, culture, etc.)
- **A statement of how many times the experiment shown was replicated in the laboratory**
- **Definitions of statistical methods and measures**: For small sample sizes (n<5) descriptive statistics are not appropriate, instead plot individual data points
  - Very common tests, such as *t*-test, simple χ^2 tests, Wilcoxon and Mann-Whitney tests, can be unambiguously identified by name only, but more complex techniques should be described in the methods section
  - Are tests one-sided or two-sided?
  - Are there adjustments for multiple comparisons?
  - **Statistical test results**, e.g., ***P* values**
  - Definition of **'center values'** as **median or mean**;
  - Definition of **error bars as s.d. or s.e.m. or c.i.**

Please ensure that the answers to the following questions are reported in the manuscript itself. We encourage you to include a specific subsection in the methods section for statistics, reagents and animal models. Below, provide the page number or section and paragraph number.

Statistics and general methods

	**Reported in section/paragraph or page #**
1. How was the sample size chosen to ensure adequate power to detect a pre-specified effect size? (Give section/paragraph or page #)	No formal statistical method was used to predetermine sample size. Sample sizes were chosen based on previous experience, published studies using similar models, and practical considerations of the animal experiments. This has
For animal studies, include a statement about sample size estimate even if no statistical methods were used.	For animal experiments, the sample size was chosen to provide sufficient biological replication while considering ethical principles for animal use.
2. Describe inclusion/exclusion criteria if samples or animals were excluded from the analysis. Were the criteria pre-established? (Give section/paragraph or page #)	No animals or samples were excluded from the analysis. Animals that successfully underwent model establishment and completed the experimental procedures were included.This has been stated in the Methods section. (Section Statistical Analysis/ page 12)
3. If a method of randomization was used to determine how samples/animals were allocated to experimental groups and processed, describe it. (Give section/paragraph or page #)	Animals were randomly assigned to the indicated groups after acclimatization. This has been stated in the Methods section (Section Murine Orthotopic Colorectal Cancer Model , page 10 ; And Section Liver metastasis model, page 11)
For animal studies, include a statement about randomization even if no randomization was used.	/

4. If the investigator was blinded to the group allocation during the experiment and/or when assessing the outcome, state the extent of blinding. (Give section/paragraph or page #)	Ex vivo bioluminescence images, gross liver photographs, and H&E sections were coded before analysis, and investigators performing quantification were blinded to group allocation. This has been described in the Methods section (Section Murine Orthotopic Colorectal Cancer
For animal studies, include a statement about blinding even if no blinding was done.	/
5. For every figure, are statistical tests justified as appropriate?	Statisti. The statistical tests used for each experimei
Do the data meet the assumptions of the tests (e.g., normal distribution)?	Data distribution was assessed prior to statistical testing. For normally distributed data, parametric tests (Student's t-test or ANOVA) were applied. For non-normally
Is there an estimate of variation within each group of data?	Data are presented as mean ± standard deviation (S
Is the variance similar between the groups that are being statistically compared? (Give section/paragraph or page #)	Variance between groups was assessed during statistical analysis and was comparable between groups in the experiments performed. This has been described in the Methods section(Section Statistical Analysis, page12)

Reagents

Reported in section/paragraph or page #

6. Report the source of antibodies (vendor and catalog number)	The sources and catalog numbers of all primary antibodies used in this study are provided in the Methods section (Section Western Blot Analysis / page 8)
7. Identify the source of cell lines and report if they were recently authenticated (e.g., by STR profiling) and tested for mycoplasma contamination	CRC cell lines (HCT116 and SW480) were obtained from the American Type Culture Collection (ATCC). Cell lines were authenticated by short tandem repeat (STR) profiling and were routinely tested for mycoplasma contamination using PCR-based assays. No mycoplasma contamination was

Animal Models

Reported in section/paragraph or page #

8. Report species, strain, sex and age of animals	BALB/c nude mice, female, 4–6 weeks old, were used in this study
9. For experiments involving live vertebrates, include a statement of compliance with ethical regulations and identify the committee(s) approving the experiments.	All animal experiments were conducted in accordance with the guidelines and regulations set forth by the Experiment Ethics Review Committee of Jiangsu Ocean University and were approved by this committee (Approval No. Jou2025051508). This information has been included in the

10. We recommend consulting the ARRIVE guidelines (*PLoS Biol.* **8**(6), e1000412,2010) to ensure that other relevant aspects of animal studies are adequately reported.

Human subjects

	Reported in section/paragraph or page #
11. Identify the committee(s) approving the study protocol.	The use of human tissue samples in this study was approved by the Ethics Committee of Ruijin Hospital, Shanghai Jiao Tong University School of Medicine (Approval No.
12. Include a statement confirming that informed consent was obtained from all subjects.	Written informed consent was obtained from all patients prior to sample collection.
13. For publication of patient photos, include a statement confirming that consent to publish was obtained.	Not applicable. No patient photographs or identifiable images were included in this study.
14. Report the clinical trial registration number (at ClinicalTrials.gov or equivalent).	Not applicable. This study does not involve a clinical trial.

15. For phase II and III randomized controlled trials, please refer to the CONSORT statement and submit the CONSORT checklist with your submission.

16. For tumor marker prognostic studies, we recommend that you follow the REMARK reporting guidelines.

Data deposition

	Reported in section/paragraph or page #
17. Provide accession codes for deposited data. Data deposition in a public repository is mandatory for:<br>a. Protein, DNA and RNA sequences<br>b. Macromolecular structures<br>c. Crystallographic data for small molecules<br>d. Microarray data	No new omics datasets were generated in this study. Publicly available datasets used in this work are accessible under the following accession numbers: GSE242332, GSE103475, GSE166555, GSE33113, and GSE227550. TCGA-COAD and GTEx data were obtained from publicly available databases.

Deposition is strongly recommended for many other datasets for which structured public repositories exist; more details on our data policy are available in the Guide to Authors. We encourage the provision of other source data in supplementary information or in unstructured repositories such as Figshare and Dryad. We encourage publication of Data Descriptors (see Scientific Data) to maximize data reuse.

18. If computer code was used to generate results that are central to the paper's conclusions, include a statement in the Methods section under "**Code availability**" to indicate whether and how the code can be accessed. Include version information as necessary and any restrictions on availability.	Data analyses were performed using R (version 4.4.2) and Seurat. Custom scripts are available upon reasonable request.
