# Supplementary material for: FOXP4 promotes metastatic progression in colorectal cancer through transcriptional activation of BAG3
Source: Cell Death Dis. 2026 Jun 11;17(1):739. doi: 10.1038/s41419-026-08941-5 (PMC13478455; doi:10.1038/s41419-026-08941-5)

FIG.3B

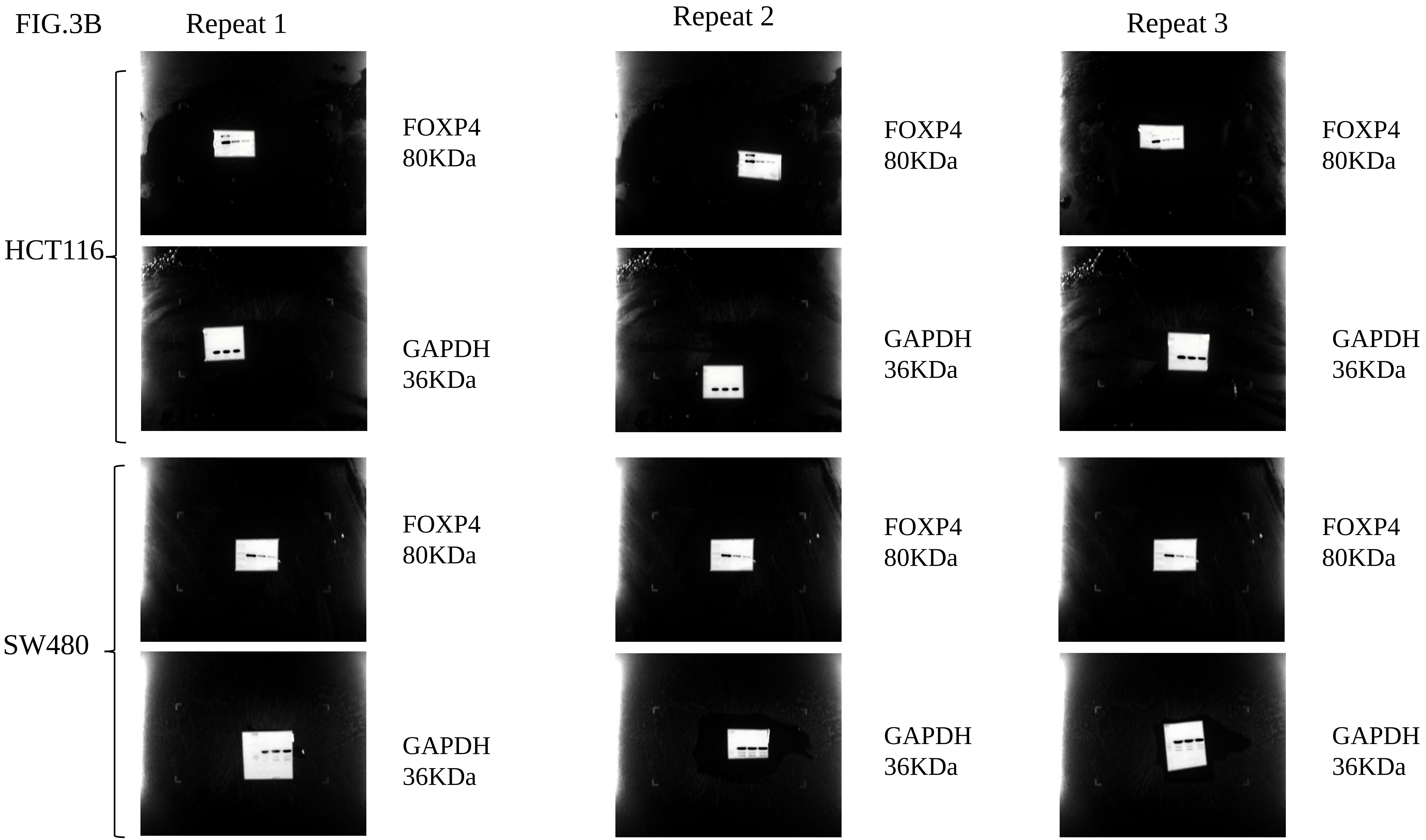
Repeat 1
Repeat 2
Repeat 3
HCT116
FOXP4
80KDa
GAPDH
36KDa
SW480
FOXP4
80KDa
GAPDH
36KDa

FIG.4E

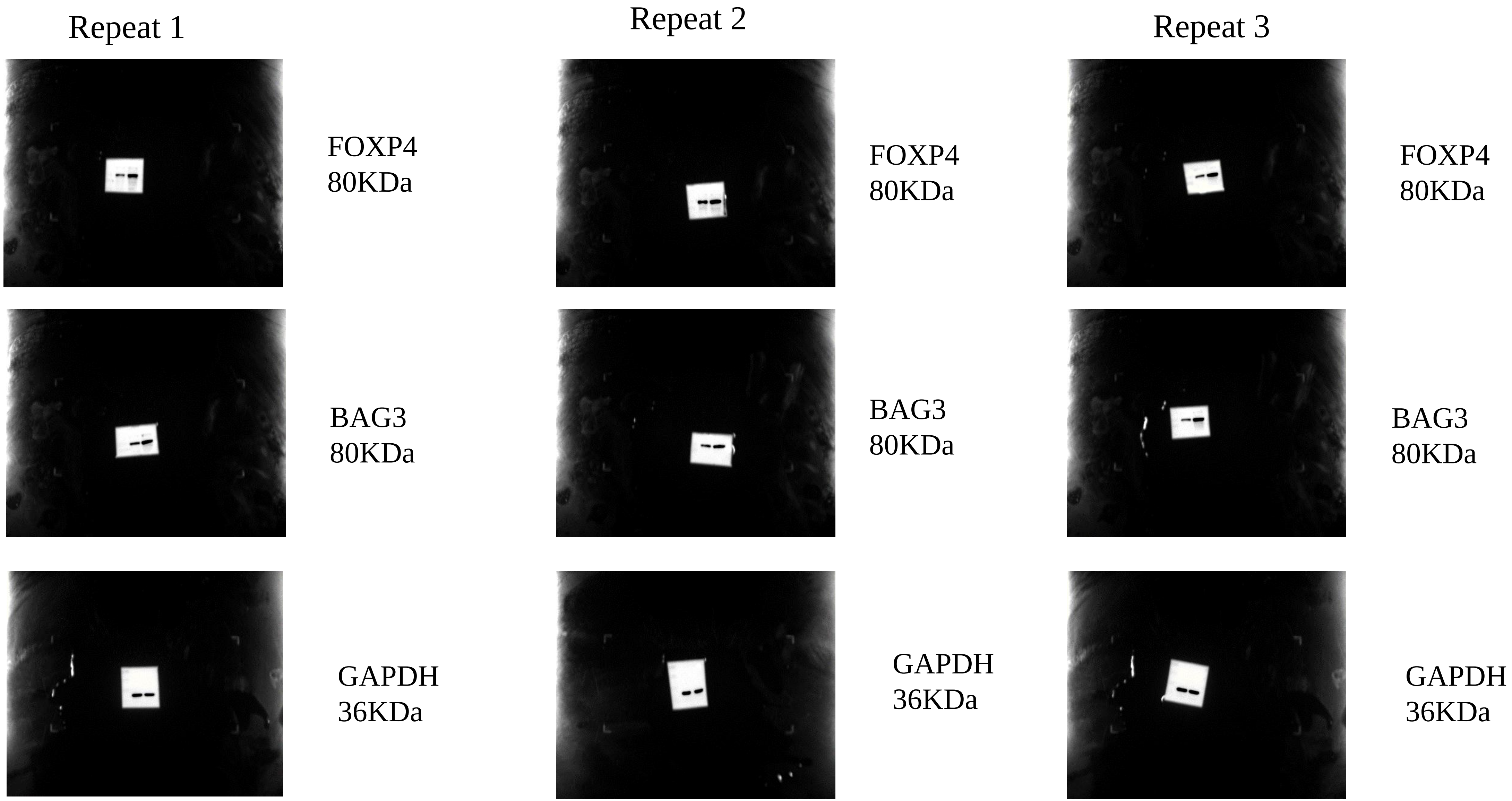
Repeat 1
Repeat 2
Repeat 3
FOXP4
80KDa
FOXP4
80KDa
FOXP4
80KDa
BAG3
80KDa
BAG3
80KDa
BAG3
80KDa
GAPDH
36KDa
GAPDH
36KDa
GAPDH
36KDa

FIG.4G

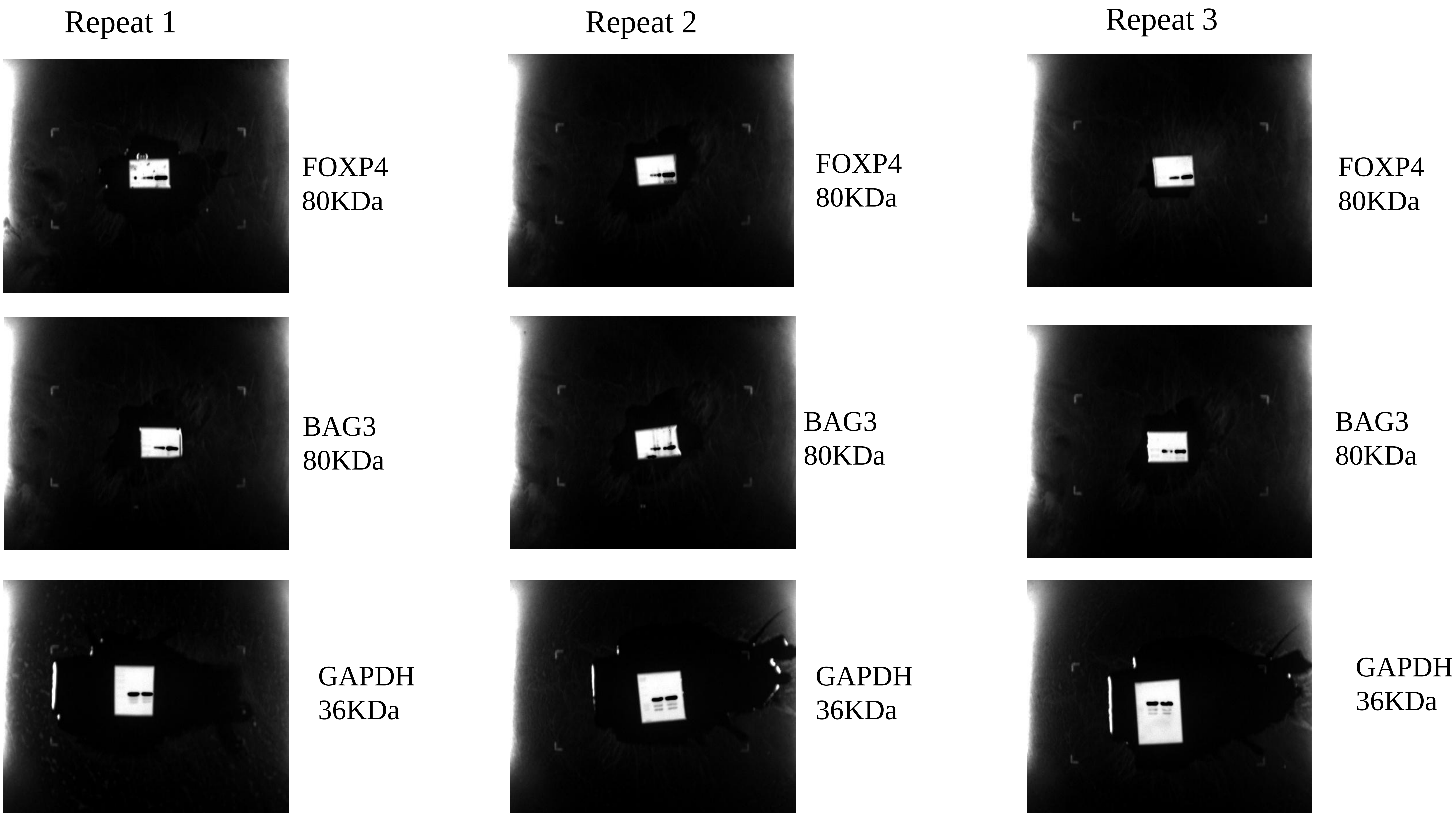

Repeat 1
Repeat 2
Repeat 3
FOXP4
80KDa
BAG3
80KDa
GAPDH
36KDa
FOXP4
80KDa
BAG3
80KDa
GAPDH
36KDa
FOXP4
80KDa
BAG3
80KDa
GAPDH
36KDa

FIG.4I

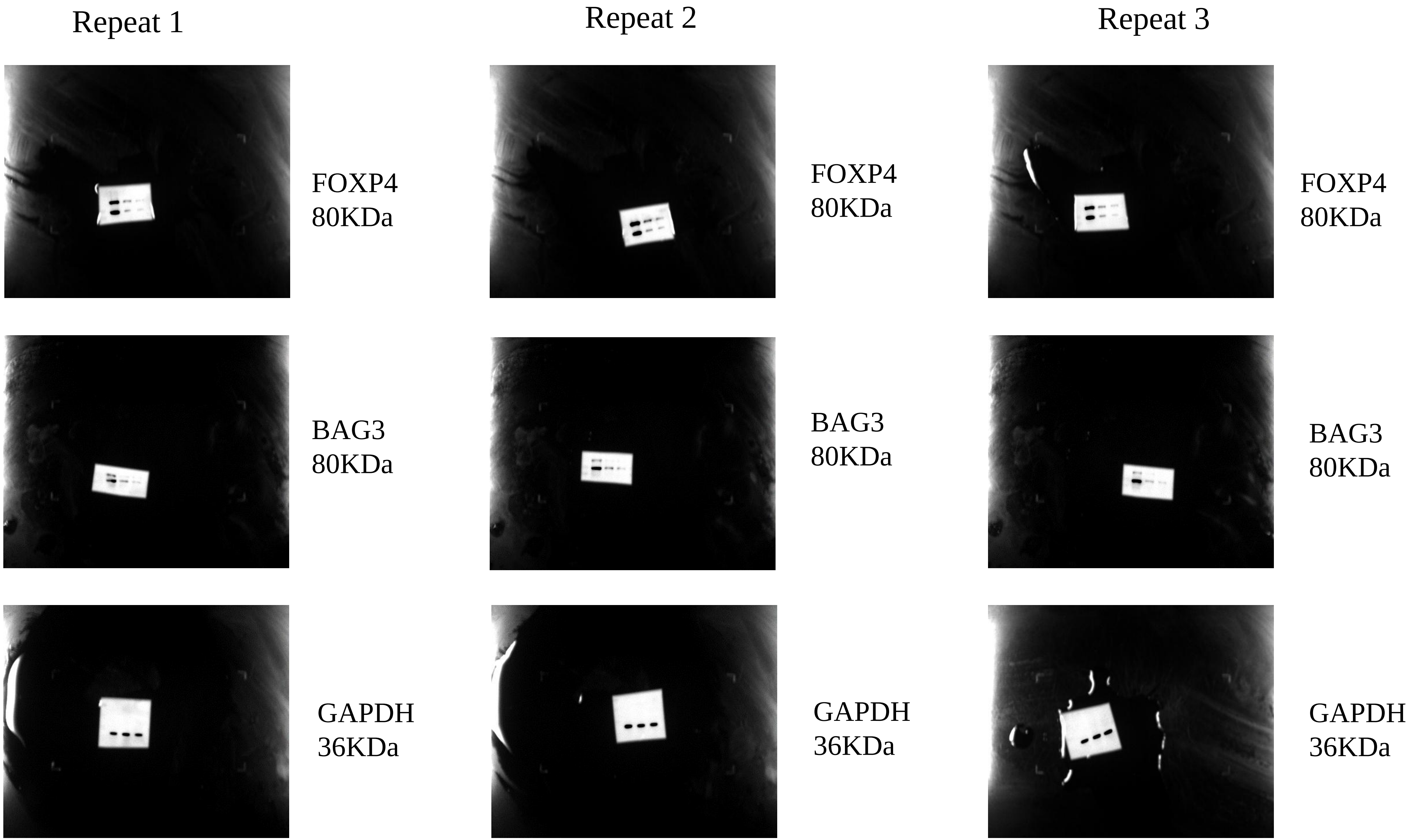
Repeat 1
Repeat 2
Repeat 3
FOXP4
80KDa
BAG3
80KDa
GAPDH
36KDa

FIG.4K

Repeat 1 Repeat 2 Repeat 3

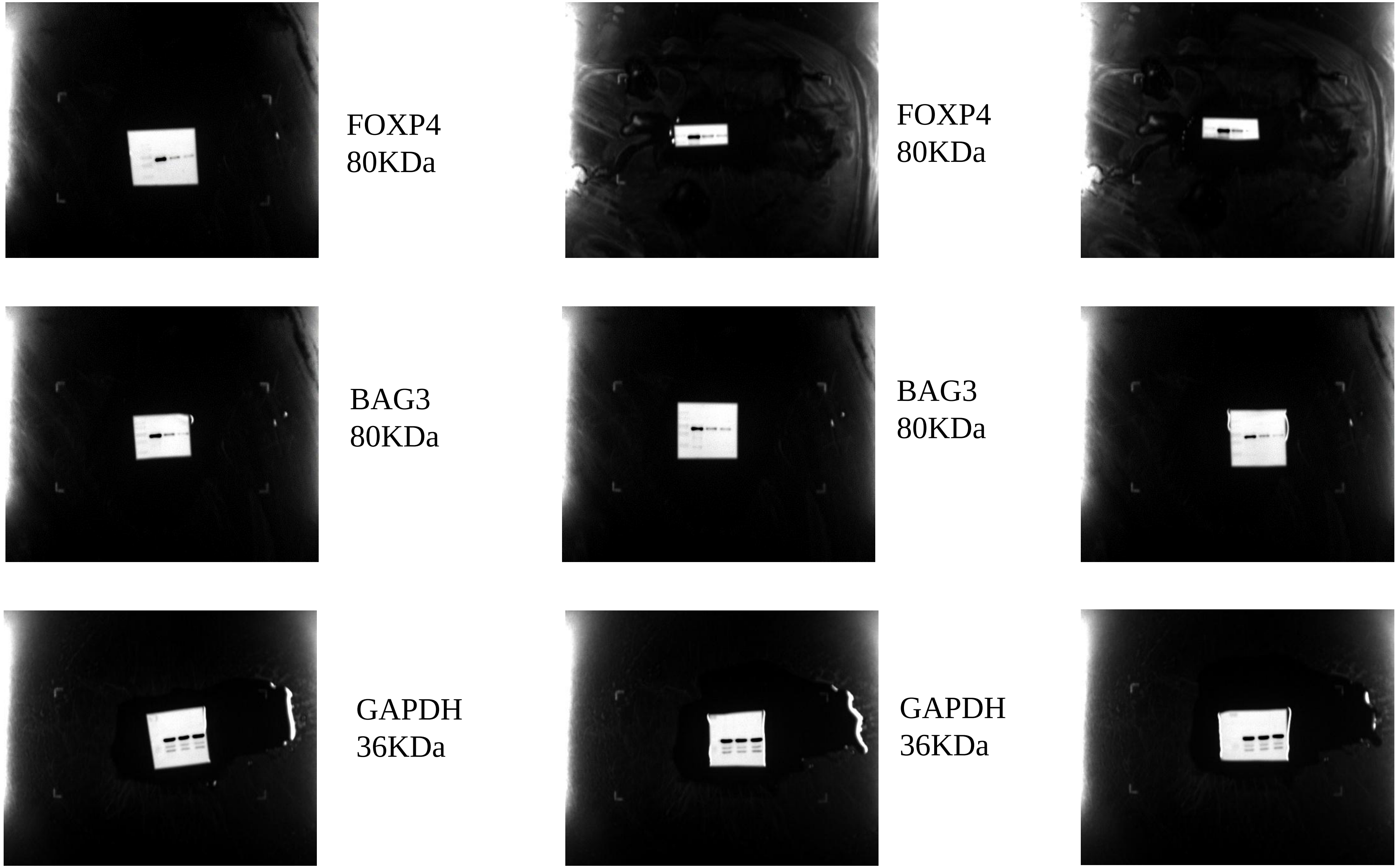

Repeat 1: FOXP4 80KDa; BAG3 80KDa; GAPDH 36KDa

Repeat 2: FOXP4 80KDa; BAG3 80KDa; GAPDH 36KDa

Repeat 3: FOXP4 80KDa; BAG3 80KDa; GAPDH 36KDa

FIG.6A

HCT116

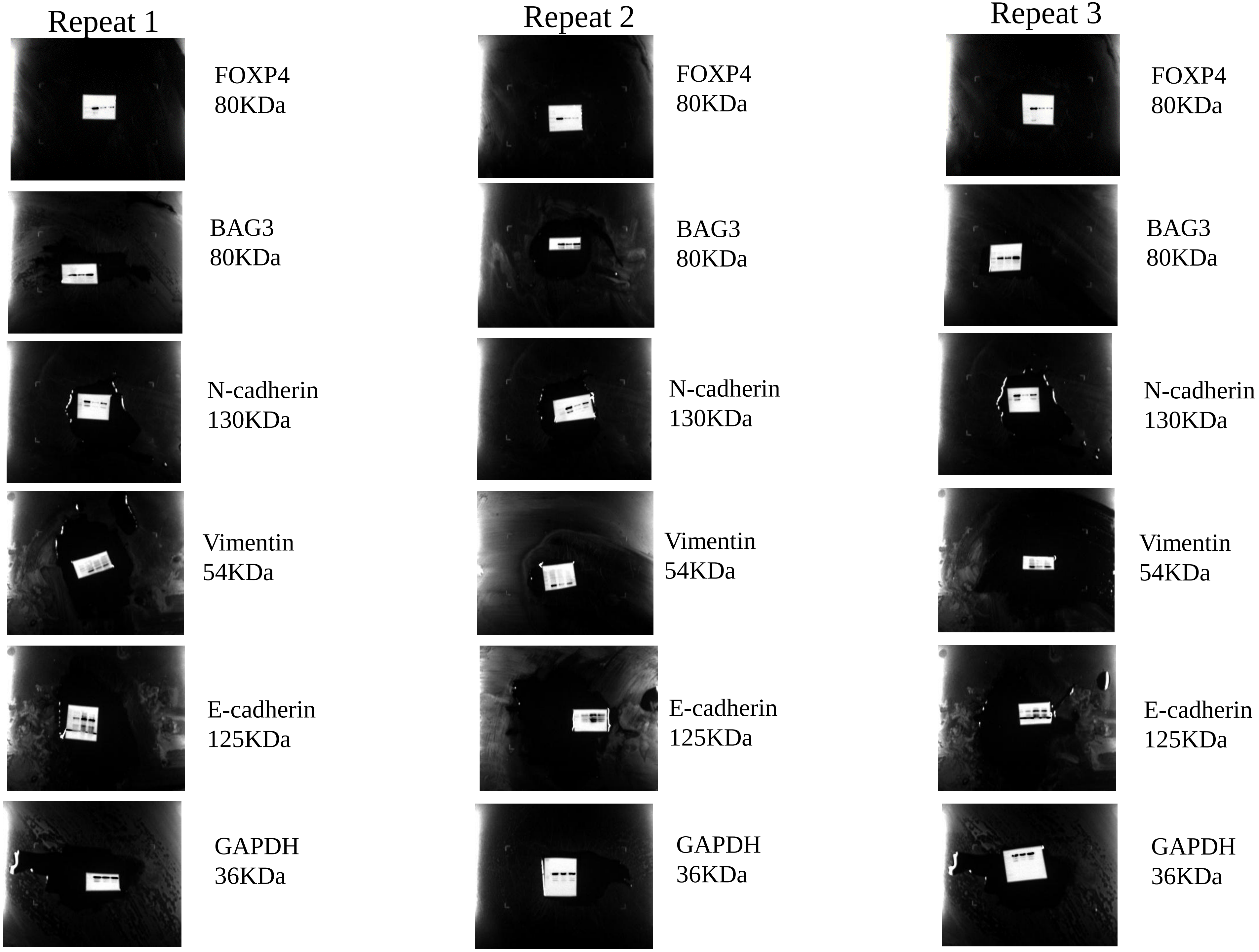

FIG.6A

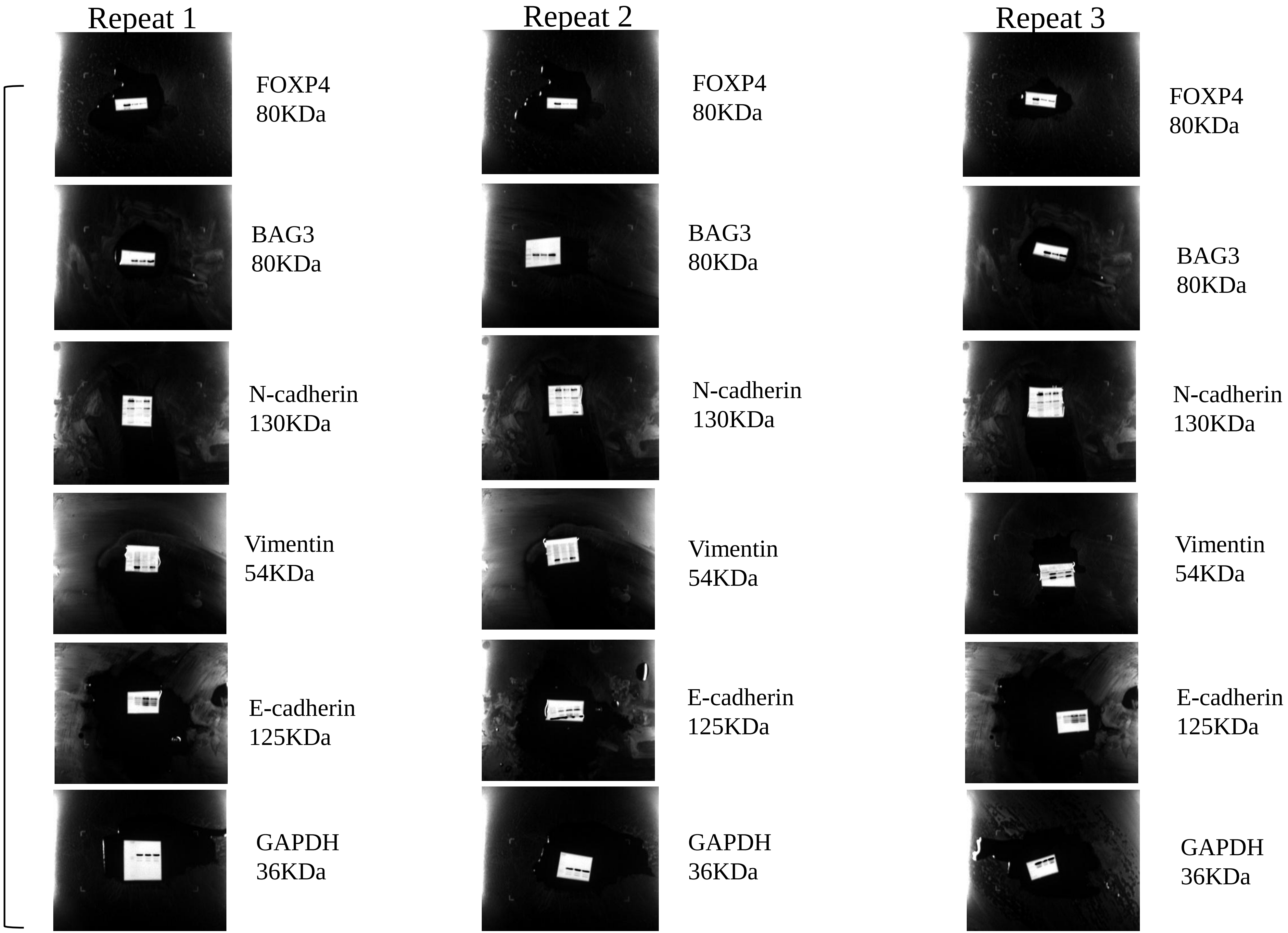
Repeat 1
Repeat 2
Repeat 3
SW480
FOXP4
80KDa
BAG3
80KDa
N-cadherin
130KDa
Vimentin
54KDa
E-cadherin
125KDa
GAPDH
36KDa

FIG.7A

HCT116

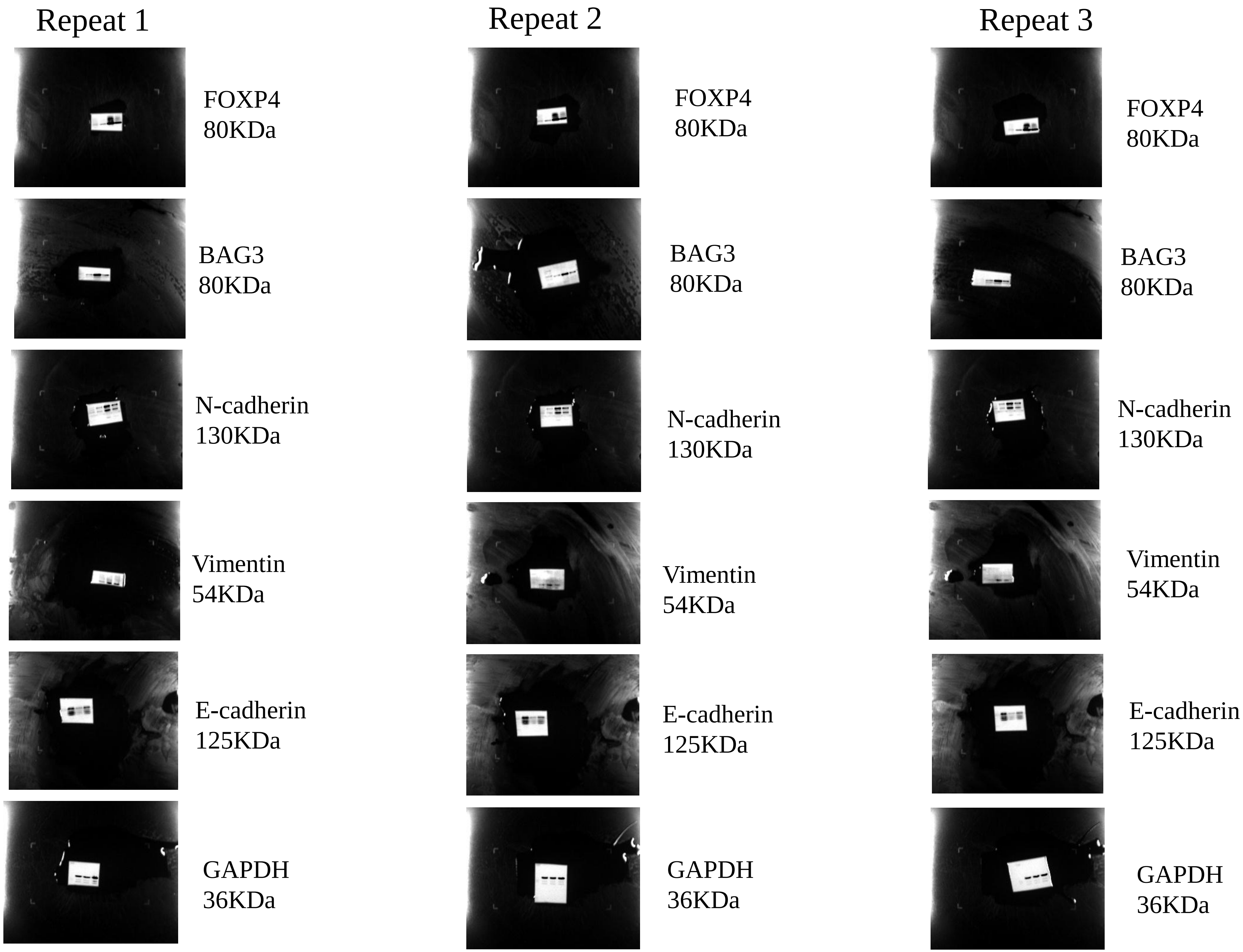

FIG.7A

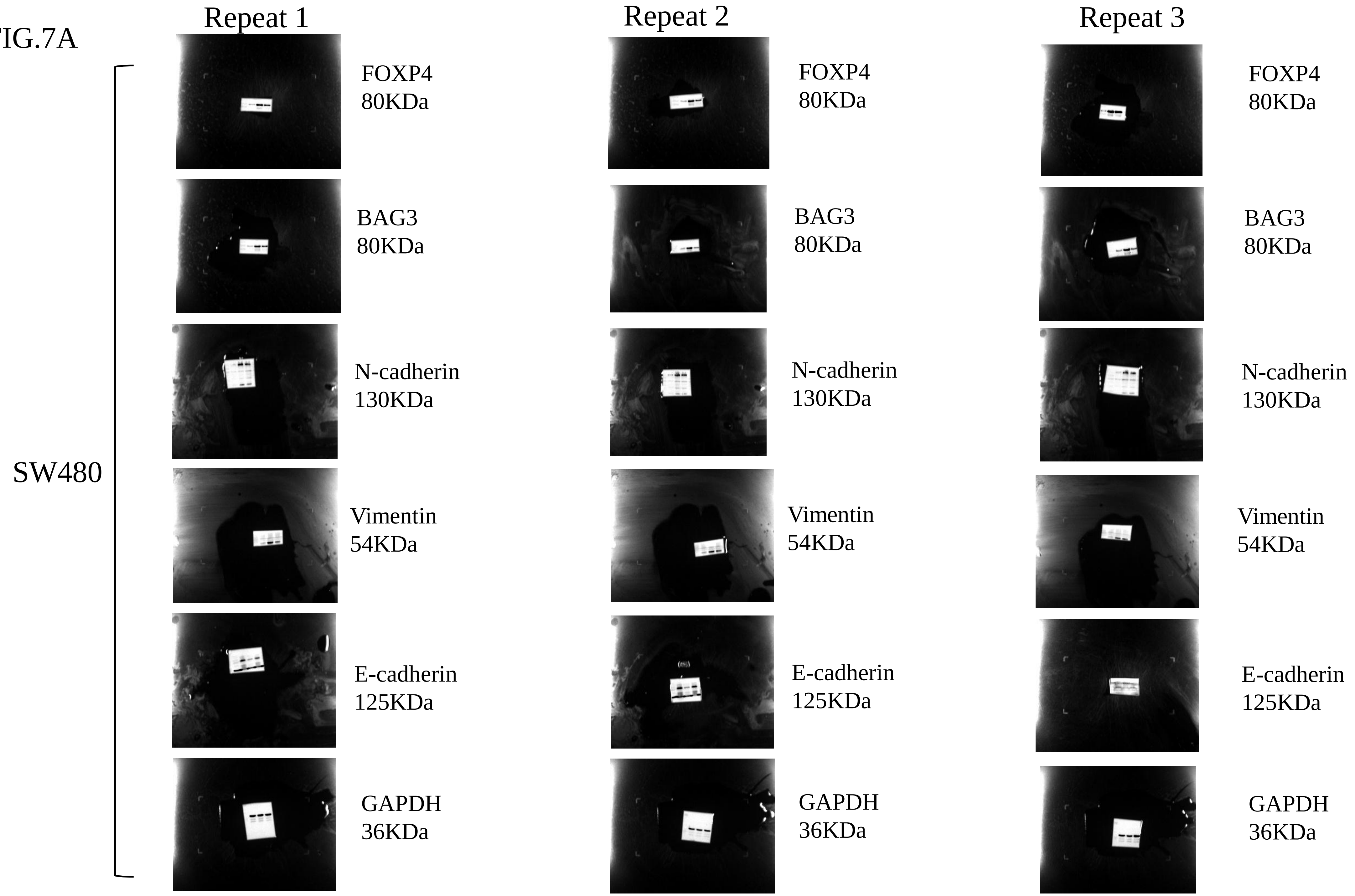
Repeat 1
Repeat 2
Repeat 3
SW480
FOXP4
80KDa
BAG3
80KDa
N-cadherin
130KDa
Vimentin
54KDa
E-cadherin
125KDa
GAPDH
36KDa

FIG.S3A

HCT116

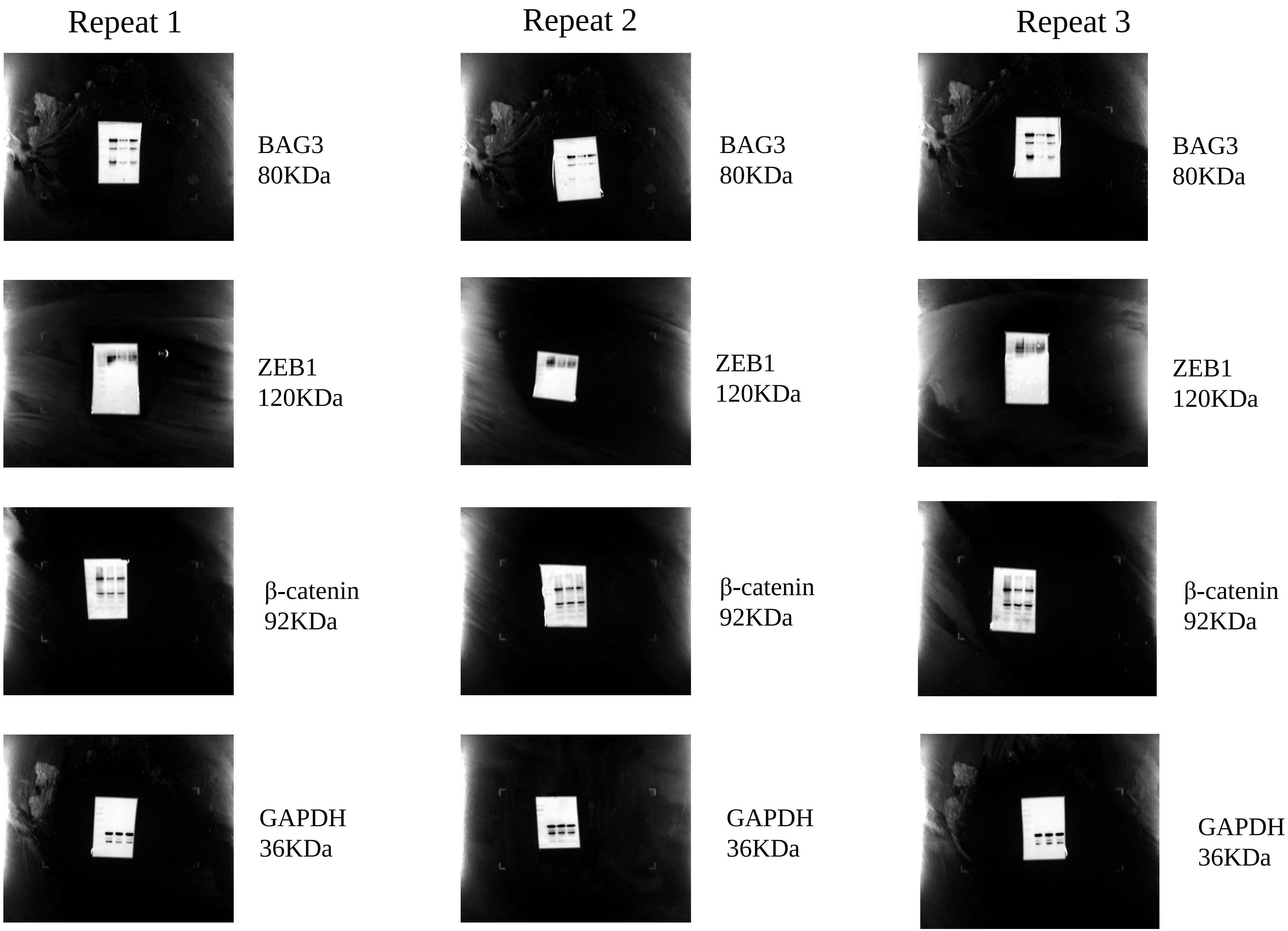

FIG.S3B

HCT116

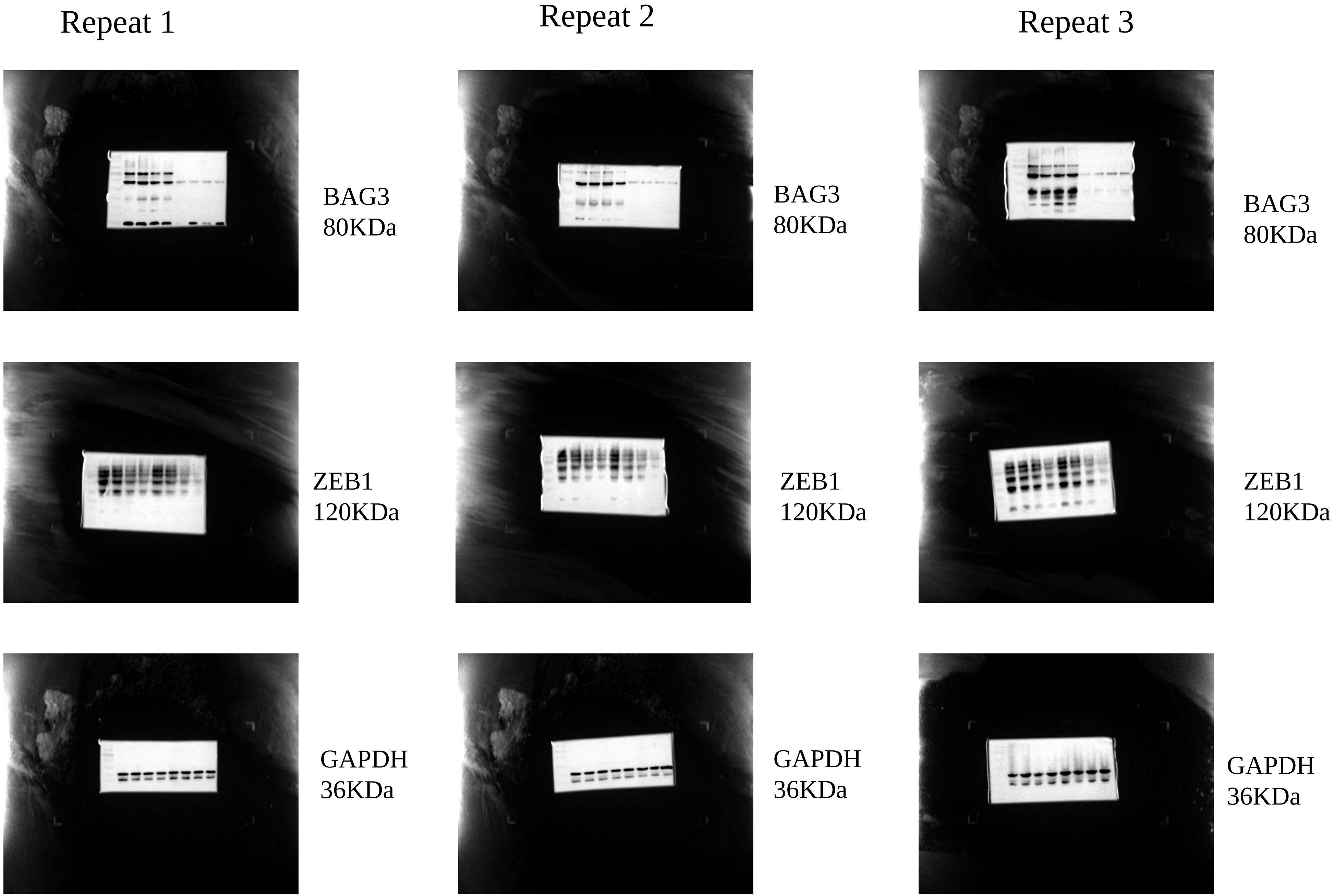

Supplement: Supplementary file 2 [file 41419_2026_8941_MOESM3_ESM.pdf]
