# Supplementary material for: FOXP4 promotes metastatic progression in colorectal cancer through transcriptional activation of BAG3
Source: Cell Death Dis. 2026 Jun 11;17(1):739. doi: 10.1038/s41419-026-08941-5 (PMC13478455; doi:10.1038/s41419-026-08941-5)
Supplement: Supplementary file 3 [file 41419_2026_8941_MOESM1_ESM.docx]

**Supplementary Figure Legends**

**Figure S1. Association of FOXP4 expression with copy-number alteration and EMT-related features.**

 **(A) Comparison of FOXP4 mRNA expression across different FOXP4 copy-number states (diploid, gain, and amplification).
(B) Spearman correlation analysis between FOXP4 expression and EMT-related signature scores in the TCGA-COAD cohort.**

**Figure S2. Representative H&E staining of liver sections showing metastatic lesions in different groups.**

**The upper panel shows the HCT116-derived liver metastasis model, and the lower panel shows the SW480-derived model. Low-magnification views are shown on the left, with corresponding magnified views on the right. Dashed boxes indicate representative metastatic areas. The PLKO group showed more extensive metastatic lesions, the #2 group showed fewer and smaller lesions, and the #2+BAG3 group exhibited a partial restoration of metastatic burden compared with the FOXP4-knockdown group.**

**Figure S3. Effects of BAG3 on ZEB1 and β-catenin expression and on ZEB1 protein stability.**

**(A) Western blot analysis of BAG3, ZEB1, and β-catenin protein expression in the PLKO group, FOXP4-knockdown group (#2), and BAG3-rescue group (#2+BAG3).**
**(B) CHX chase assay showing changes in ZEB1 protein stability in the shNC and shBAG3 groups. Cells were treated with cycloheximide (CHX) and harvested at 0, 2, 4, and 6 h for Western blot analysis of BAG3, ZEB1, and GAPDH.**

**Figure S4. Single-cell transcriptomic analysis supports the epithelial-specific relevance of the FOXP4–BAG3 axis.**

(A) Correlation between FOXP4 and BAG3 expression in malignant epithelial cells from colorectal cancer single-cell RNA-sequencing data (GSE166555). Spearman correlation analysis showed a significant positive association (r = 0.40, p < 4.6 × 10^-41^).

(B) Comparison of EMT module scores between FOXP4-high and FOXP4-low malignant epithelial cells. Cells with high FOXP4 expression exhibited significantly elevated EMT scores (Wilcoxon test, p = 0.0107).
